# Shell neurons of the master circadian clock coordinate the phase of tissue clocks throughout the brain and body

**DOI:** 10.1186/s12915-015-0157-x

**Published:** 2015-06-23

**Authors:** Jennifer A. Evans, Ting-Chung Suen, Ben L. Callif, Andrew S. Mitchell, Oscar Castanon-Cervantes, Kimberly M. Baker, Ian Kloehn, Kenkichi Baba, Brett J. W. Teubner, J. Christopher Ehlen, Ketema N. Paul, Timothy J. Bartness, Gianluca Tosini, Tanya Leise, Alec J. Davidson

**Affiliations:** Department of Biomedical Sciences, Marquette University, Milwaukee, WI 53233 USA; Department of Neurobiology, Morehouse School of Medicine, Atlanta, GA 30310 USA; Department of Pharmacology, Morehouse School of Medicine, Atlanta, GA 30310 USA; Department of Biology and Center for Obesity Reversal, Georgia State University, Atlanta, GA 30302 USA; Department of Mathematics and Statistics, Amherst College, Amherst, MA 01002 USA; Department of Developmental Neurobiology, St. Jude Children’s Research Hospital, Memphis, TN 38105 USA

**Keywords:** Circadian, Coupling, Peripheral and central oscillators, Photoperiod, Suprachiasmatic nucleus

## Abstract

**Background:**

Daily rhythms in mammals are programmed by a master clock in the suprachiasmatic nucleus (SCN). The SCN contains two main compartments (shell and core), but the role of each region in system-level coordination remains ill defined. Herein, we use a functional assay to investigate how downstream tissues interpret region-specific outputs by using *in vivo* exposure to long day photoperiods to temporally dissociate the SCN. We then analyze resulting changes in the rhythms of clocks located throughout the brain and body to examine whether they maintain phase synchrony with the SCN shell or core.

**Results:**

Nearly all of the 17 tissues examined in the brain and body maintain phase synchrony with the SCN shell, but not the SCN core, which indicates that downstream oscillators are set by cues controlled specifically by the SCN shell. Interestingly, we also found that SCN dissociation diminished the amplitude of rhythms in core clock gene and protein expression in brain tissues by 50–75 %, which suggests that light-driven changes in the functional organization of the SCN markedly influence the strength of rhythms in downstream tissues.

**Conclusions:**

Overall, our results reveal that body clocks receive time-of-day cues specifically from the SCN shell, which may be an adaptive design principle that serves to maintain system-level phase relationships in a changing environment. Further, we demonstrate that lighting conditions alter the amplitude of the molecular clock in downstream tissues, which uncovers a new form of plasticity that may contribute to seasonal changes in physiology and behavior.

**Electronic supplementary material:**

The online version of this article (doi:10.1186/s12915-015-0157-x) contains supplementary material, which is available to authorized users.

## Background

Daily rhythms in mammalian behavior and physiology are programmed by a system of biological clocks that oscillate with a period of approximately 24 h [1]. At the cellular level, self-sustained circadian rhythms are generated by interconnected feedback loops involving the transcription of clock genes (e.g., *Period1* (*Per1*), *Period2* (*Per2*), *Cryptochrome1* (*Cry1*), *Cryptochrome2* (*Cry2*)) whose protein products inhibit their own transcription once every 24 h [2]. Although this molecular clock exists in nearly every cell of the body, a central pacemaker within the suprachiasmatic nucleus (SCN) is needed to coordinate these clocks with one another and the solar day [1]. The SCN synchronizes downstream tissues through a variety of pathways, including direct synaptic connections, release of humoral factors, and via control of overt rhythms such as locomotion, body temperature, feeding, and hormone release [3]. This complexity in SCN outputs has made it difficult to precisely map the functional connections between the SCN and downstream tissues. Deeper understanding of how the SCN communicates with downstream tissues would provide critical insight into the functional organization of the circadian system, which may reveal novel ways to manipulate temporal coordination in the circadian system and alleviate the adverse health consequences of shift work and jetlag [4].

With regards to system-level organization, a fundamental question that remains unresolved is whether time-of-day cues are transmitted by a specific subclass of SCN neuron. The SCN is a network of neural oscillators organized into two functionally distinct compartments (i.e., SCN shell and core) distinguished by connectivity, gene expression, neuropeptide expression, and responses to environmental stimuli [5–10]. Although species differences exist, the SCN shell contains a dense population of arginine vasopressin (AVP) neurons, and the SCN core contains vasoactive intestinal polypeptide (VIP) neurons [11]. Neuroanatomical studies indicate that both AVP and VIP neurons form connections with proximal targets in the brain, often projecting to the same structure [5, 12]. These neuroanatomical studies provide insight into SCN efferent pathways, but it remains unclear if the outputs from SCN shell and core are redundant or functionally distinct. Because the SCN can communicate with target tissues through non-synaptic mechanisms (e.g., paracrine signaling, control of overt rhythms) [3], addressing the import of outputs from different SCN regions requires a functional assay that can detect the downstream consequences of cues transmitted by each SCN compartment. Herein, we develop a functional assay to identify the SCN compartment that provides time-of-day cues to downstream tissues, based on previous work demonstrating that a clock maintains a stable phase relationship with its synchronizing stimulus. Extending this logic to the entraining influence of the SCN, we can infer a functional relationship when a given tissue maintains phase synchrony with a specific SCN compartment. The strength of this approach is that it does not require direct synaptic connections, nor does it require that tissues express rhythms that are in phase with the SCN.

One difficulty in applying this approach is that neurons in the SCN shell and core typically display synchronized rhythms. Because regional activity patterns usually overlap in time, this limits the ability to distinguish how downstream tissues interpret outputs from a specific SCN compartment. However, rhythms of SCN shell and core neurons can be dissociated by manipulating environmental lighting conditions *in vivo*. For example, chronic exposure to constant light (LL) “splits” the activity rhythm in nocturnal rodents. LL-induced split rhythms at the behavioral level correspond to anti-phase rhythms between the left and right SCN [13–16], as well as anti-phase rhythms between the shell and core within each SCN lobe [13, 16]. In LL-induced split hamsters, it has been shown that the lateral subparaventricular zone tracks the phase of the SCN shell and the paraventricular nucleus tracks the phase of the SCN core, which suggests that outputs from these SCN regions are functionally distinct and tissue specific [13]. However, the SCN region providing cues to these downstream tissues remains unclear because LL does not produce a clear dissociation of SCN shell and core, instead causing the SCN core to cycle in phase with the contralateral shell (i.e., in the opposite SCN lobe) [13–15]. Further, because *c-fos* was used as an index of connectivity, the relevance of these region-specific changes for molecular clock function in downstream tissues is not clear. Complementary studies using non-24 h light:dark cycles to dissociate the SCN shell and core of the rat also indicate that both SCN compartments contribute to the expression of overt rhythms, with the phase of rapid eye movement sleep and some hormonal rhythms dictated specifically by the SCN shell [17–20]. However, changes in the function of downstream clocks have yet to be examined under non-24 h light:dark cycles or LL. Collectively, these studies suggest that the SCN shell and core differ in their contribution to system-level coordination, but it remains an open question whether SCN outputs derive from a specific compartment or whether each compartment provides cues in a tissue-specific or functionally-distinct manner.

In addition to the exotic lighting conditions described above, the SCN can be dissociated using more naturalistic environmental manipulations such as simulated seasonal changes in day length [21–23]. We have recently demonstrated that *in vivo* exposure to long, summer-like day lengths causes the SCN shell and core to cycle out of phase in mice [21]. Use of seasonal changes in day length presents a unique opportunity to examine the functional role of outputs from each SCN compartment because the system is stably entrained and under strict experimental control. Furthermore, there is less variability among individual mice in the timing and pattern of network reorganization relative to other dissociation protocols. Finally, because SCN dissociation through photoperiodic manipulations temporally reorganizes the SCN core and shell, but not the left and right SCN, this approach can be used to more precisely establish the neuroanatomical source of the cues. Herein, we take advantage of SCN dissociation under long day lengths to investigate the functional role of SCN shell and core outputs. After inducing SCN dissociation with long day lengths, we assessed whether molecular rhythms in tissues throughout the brain and body maintain phase synchrony with the SCN shell or the SCN core. Our results indicate that SCN compartments differ in their functional contribution to system-level coordination, with the SCN shell setting the phase of nearly all of the 17 tissues studied here. These data demonstrate that the function of outputs from different SCN compartments is highly specialized, which serves to maintain timing among clocks in the brain and body in a changing environment. Although the SCN core does not appear to regulate the phase of downstream oscillators, we find evidence that the integrated function of the SCN is critically important for maintaining the amplitude of the molecular clock in downstream tissues. Photoperiodic modulation of molecular rhythm amplitude in downstream tissues reveals a new form of plasticity that may encode time of year at the level of the local clock.

## Results

### Long day lengths dissociate rhythms in the SCN shell and core

**Fig. 1:**
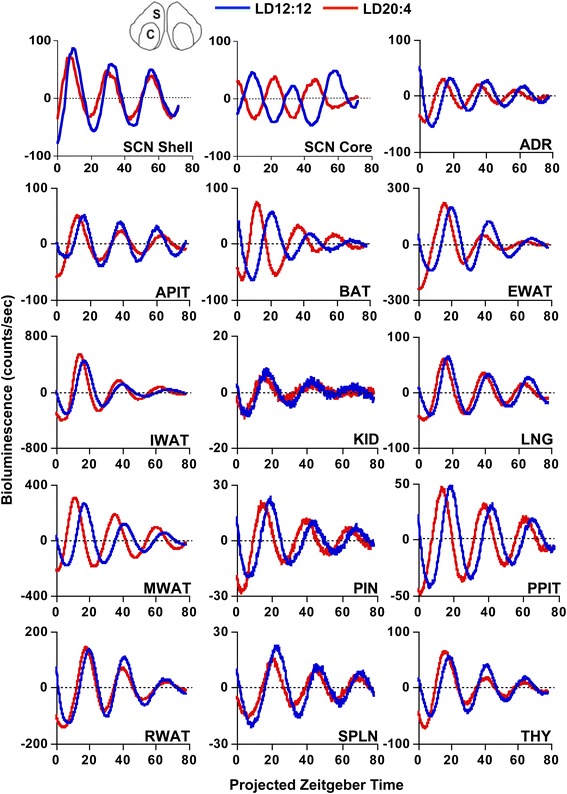
Representative time series of bioluminescence rhythms measured *in vitro* from tissues collected from PER2::LUC mice housed under LD12:12 or LD20:4. Time series are de-trended and corrected for the Zeitgeber Time (ZT) of dissection. SCN shell and SCN core regions used for analyses are illustrated at the top of the figure (S and C, respectively). ADR, Adrenal gland; APIT, Anterior pituitary gland; BAT, Brown adipose tissue; EWAT, Epididymal white adipose tissue; IWAT, Inguinal white adipose tissue; KID, Kidney; LNG, Lung; MWAT, Mesenteric white adipose tissue; PIN, Pineal gland; PPIT, Posterior pituitary gland; RWAT, Retroperitoneal white adipose tissue; SPLN, Spleen; THY, Thymus

**Fig. 2:**
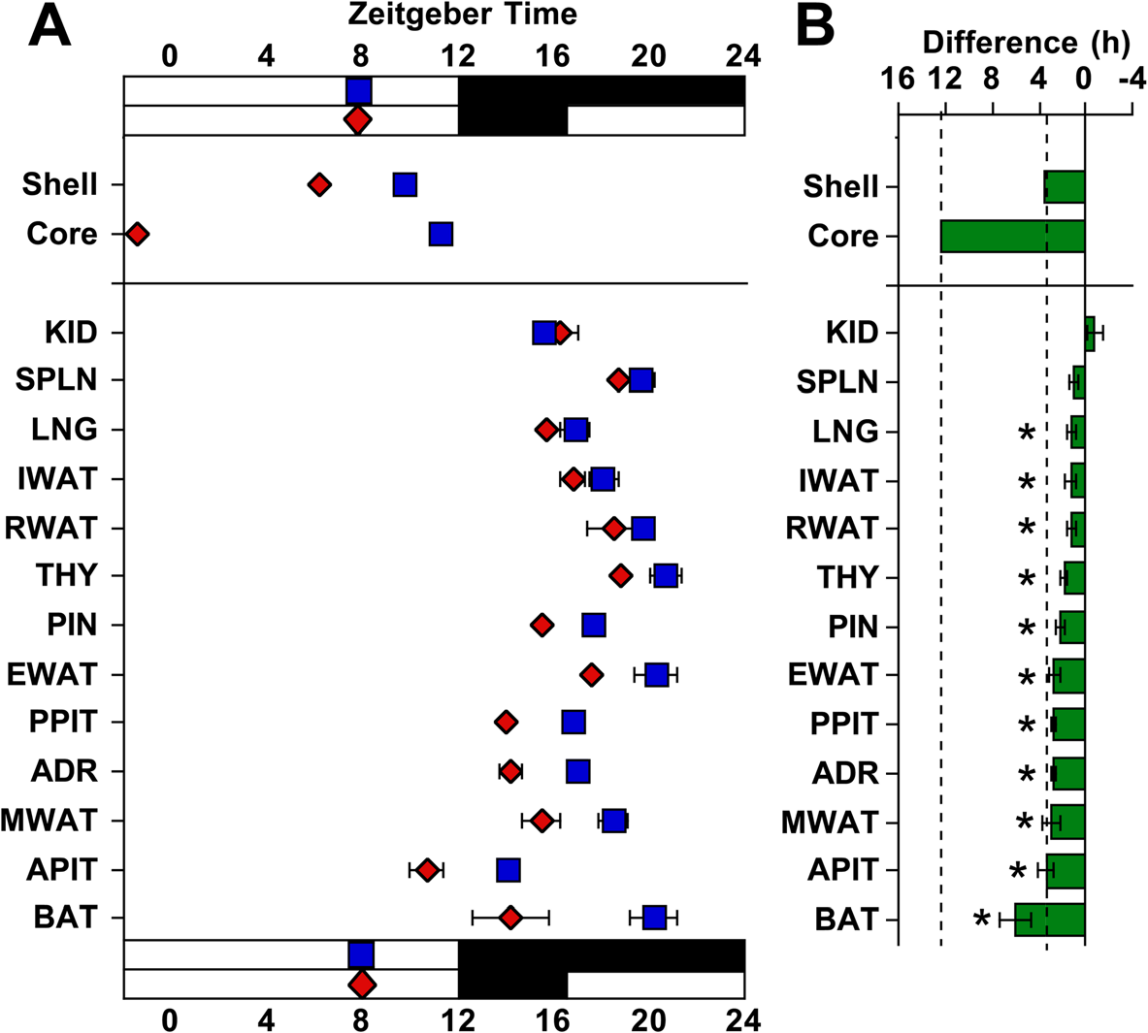
The phase of PER2::LUC rhythms in non-SCN tissues is shifted under long photoperiods by the SCN shell. **a** Time of peak bioluminescence (± SEM) on the first cycle *in vitro* displayed by SCN regions and peripheral tissues collected from PER2::LUC mice housed under LD12:12 (blue symbols) or LD20:4 (red symbols). The white and black bars on the abscissa represent lighting conditions for each photoperiod, with internal symbols indicating the time of dissection. *n* = 5–14/tissue/photoperiod. **b** Difference in the peak time of PER2::LUC expression (± SEM) between cultures collected under LD12:12 and LD20:4. Tissues are ordered by the magnitude of the difference in peak time. Dashed vertical lines indicate the magnitude of the shift displayed by the SCN shell and SCN core. *Significant phase shift different from 0 h, one sample *t*-test, *P* <0.05

Male PERIOD2::LUCIFERASE (PER2::LUC) mice [24] were housed under a standard lighting condition with 12 h of light (LD12:12) or a long day with 20 h of light (LD20:4), which produced re-entrainment of both locomotor activity and sleep rhythms (Additional file 1: Figure S1). To confirm photoperiodic reorganization of the SCN network, SCN slices were collected for real-time bioluminescence imaging of PER2::LUC rhythms. Consistent with our previous work [21], LD20:4 temporally reorganized the SCN network through region-specific shifts in the phase of PER2::LUC rhythms (Figs. 1 and 2). Relative to LD12:12, *in vivo* exposure to LD20:4 caused both the SCN shell and core to display an earlier time of peak PER2::LUC expression on the first cycle *in vitro.* The SCN shell and core, however, displayed differential responses (Fig. 1), with the SCN shell advancing by approximately 3.5 h and the SCN core shifting by approximately 12 h. These results confirm that *in vivo* exposure to long day lengths dissociates the SCN shell and core, which can be used to assess the functional role of region-specific communication with downstream tissues.

### The SCN shell sets the phase of peripheral clocks

PER2::LUC rhythms were evident in a wide range of central and peripheral structures, with tissue-specific differences in peak time, period, amplitude, and damping rate *in vitro* (Figs. 1 and 2, Additional file 1: Table S1). First we assessed whether the phase of PER2::LUC rhythms *in vitro* is a faithful reflection of *in vivo* phase by collecting tissues from LD12:12 mice either 2 h before lights-off or 2 h after lights-on. By assessing the effect of dissections performed at different times of day, this test determines whether the phase of PER2::LUC rhythms *in vitro* is reset by the culture procedure itself [25]. The majority of peripheral tissues were unaffected by dissection, with only 5/18 affected by the timing of the culture procedure (Additional file 1: Figure S2). The only peripheral tissues completely reset by dissection were the three ocular tissues (cornea, retina, retinal pigmented epithelium), although the liver and esophagus were also shifted (Additional file 1: Figure S2). For tissues not reset by dissection, dissection did not produce systematic effects on other rhythmic parameters, including amplitude and period of PER2::LUC rhythms (Additional file 1: Table S1). The relatively low number of tissues affected by the culture procedure supports the use of *ex vivo* real-time bioluminescence luminometry to assess *in vivo* photoperiodic changes in peripheral clock function. Further, these results demonstrate the utility of PER2::LUC assays to investigate a wide variety of peripheral tissues that may be of interest in circadian and non-circadian research.

To test for photoperiodic changes in downstream oscillators, tissues not reset by dissection were collected from LD12:12 and LD20:4 mice 2–4 h before lights-off (Figs. 1 and 2, Additional file 1: Table S2). After exposure to LD20:4, nearly all peripheral tissues exhibited a 2–4 h advance in the time of peak PER2::LUC, which was similar in magnitude to the 3 h shift displayed by the SCN shell (Figs. 1 and 2). In contrast, none of the peripheral tissues displayed a large 12 h advance like that displayed by the SCN core (Figs. 1 and 2), suggesting that this specific SCN compartment does not set the phase of these tissues. The consistency of these results is highlighted by the variety of systems examined here, including numerous endocrine glands (adrenal gland, anterior pituitary, pineal gland, posterior pituitary, and thymus) and several different adipose depots (epididymal white adipose, mesenteric white adipose, and brown adipose). Overall, this pattern of results demonstrates that peripheral tissues maintain phase synchrony with the SCN shell, which indicates that they receive time-of-day cues from this specific SCN compartment.

**Fig. 3:**
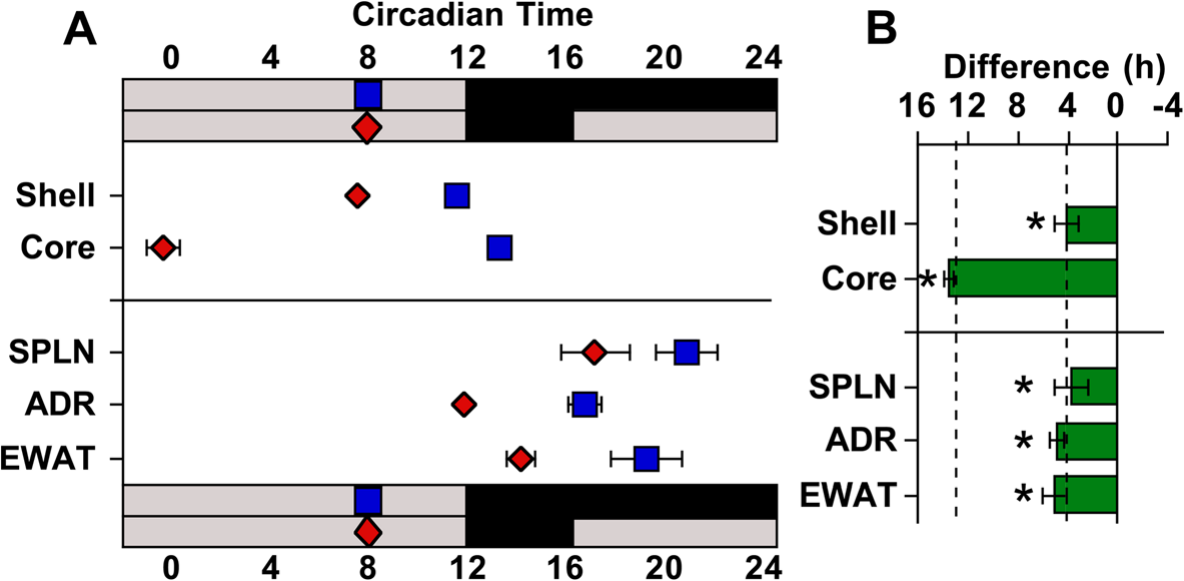
Photoperiodic changes in the phase of peripheral tissues persist after release into constant darkness. **a** Time of peak bioluminescence (± SEM) on the first cycle *in vitro* displayed by SCN regions and peripheral tissues collected after release into constant darkness from LD12:12 (blue symbols) or LD20:4 (red symbols). *n* = 3/photoperiod for SCN, *n* = 6/photoperiod for peripheral tissues. **b** Summary plots of photoperiod-induced changes in the phase of peripheral tissues after release into constant darkness. Tissues are ordered by the magnitude of the difference in peak time. Dashed vertical lines indicate the magnitude of the shift displayed by the SCN shell and SCN core after release into constant darkness. *Significant phase shift different from 0 h, one sample *t*-test, *P* <0.05

Because overt behavior and molecular rhythms in some peripheral tissues can be influenced directly by light [26–30], light exposure under LD20:4 may determine the phase of peripheral tissues. To test whether photoperiodic changes were due to light-induced masking, mice were released from LD12:12 or LD20:4 into constant darkness for 1 day before collection of peripheral tissues (adrenal, epididymal white adipose tissue, and spleen). This acute release into constant darkness was designed to eliminate the masking effects of light, but maintain SCN dissociation and prevent the resynchronization of SCN shell and core that occurs with longer exposure to constant darkness [21]. Consistent with this previous work, the SCN shell and core remained dissociated after 1 day in constant darkness (Fig. 3). Further, peripheral tissues continued to exhibit a phase advance similar in magnitude to that displayed by the SCN shell (Fig. 3), which indicates that light is not directly driving photoperiodic changes in the phase of peripheral clocks.

### The SCN shell sets the phase of central clocks

**Fig. 4:**
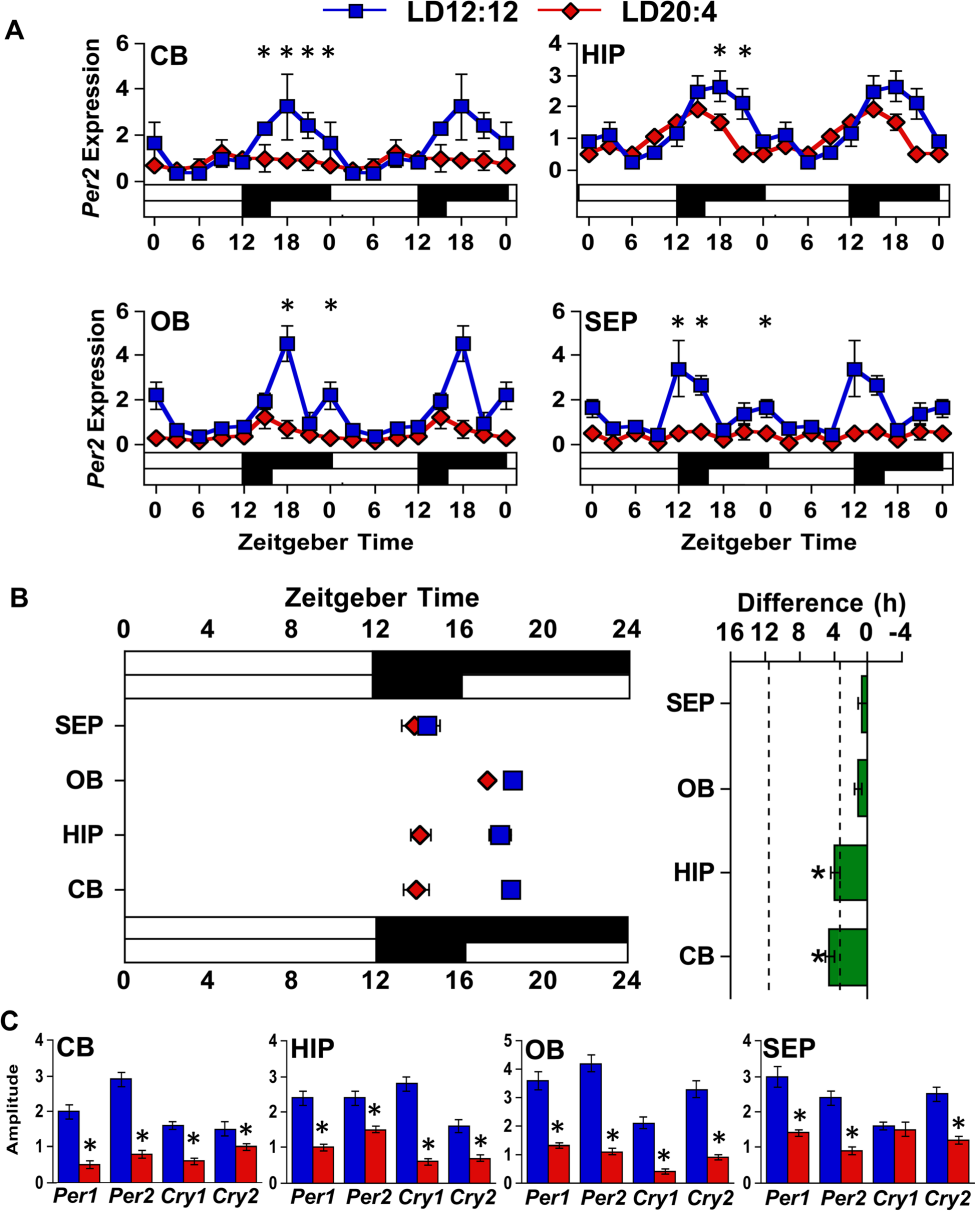
The phase and amplitude of clock gene rhythms in central tissues is influenced by photoperiod. **a** Double-plotted rhythms in *Per2* mRNA expression were measured with qRT-PCR for the cerebellum (CB), hippocampus (HIP), olfactory bulb (OB), and septum (SEP) under LD12:12 (blue symbols) and LD20:4 (red symbols). White and black bars on the abscissa represent lighting conditions. *n* = 3/time-point/photoperiod. *LD12:12 versus LD20:4, LS Means Contrasts, *P* <0.006. Cosinor analyses of Per2 rhythms are shown in Additional file 1: Table S3. **b** Summary plots of photoperiod-induced changes in the phase of *Per2* rhythms in central tissues. Tissues are ordered by the magnitude of the difference in peak time. Dashed vertical lines indicate the magnitude of the shift displayed by the SCN shell and SCN core *in vivo*, as detected with PER2 immunohistochemistry (c.f., Additional file 1: Table S4). *Significant phase shift different from 0 h, one sample *t*-test, *P* <0.05. **c** Amplitude of core clock gene expression is reduced in all four tissues. *Student’s *t*-test, *P* <0.05. Clock gene rhythms for all four tissues are illustrated in Additional file 1: Figure S3

Proximal targets in the brain receive direct projections from both SCN shell and core neurons, with tissue-specific differences in the density of innervation from each region [5]. This raises the possibility that central clocks may be distinct from peripheral tissues in that they receive time-of-day cues from each SCN region in a tissue-specific manner. Because PER2::LUC rhythms in central tissues can be reset by the culture procedure [31, 32], more traditional techniques were required to address this question. Thus, to test whether photoperiodic reorganization of the SCN influences the phase of central tissues in a manner similar to peripheral tissues, brains were collected from LD12:12 and LD20:4 mice at eight time-points spanning the circadian cycle. Cerebellum, hippocampus, olfactory bulb, and septum were collected for qRT-PCR analyses of *Per2* rhythms. To assess photoperiodic changes, cosinor analyses were used to evaluate whether daily fluctuations in *Per2* expression were rhythmic and to determine the center of gravity of clock gene expression patterns (Additional file 1: Table S3). Under LD12:12, each tissue displayed significant *Per2* rhythms with peak expression during the dark phase (Fig. 4, Additional file 1: Table S3). Under LD20:4, none of these central tissues shifted in a manner similar to the SCN core. Rather, the cerebellum and hippocampus displayed a 3 h advance of the *Per2* rhythm, while the olfactory bulb and septum did not shift significantly. Overall, these results support the conclusion that downstream oscillators are reset by time-of-day cues controlled specifically by the SCN shell, which is similar to conclusions based on PER2::LUC rhythms in peripheral tissues.

### SCN dissociation diminishes rhythms in central clocks

Unexpectedly, SCN reorganization was found to be associated with a 50–75 % reduction in the amplitude of *Per2* rhythms in central targets (Fig. 4, Additional file 1: Table S3). This large reduction in *Per2* amplitude was evident in each of the four tissues, although its magnitude varied by tissue (Fig. 4a, Additional file 1: Table S3). Interestingly, *Per2* amplitude was consistently reduced across all four tissues regardless of whether the tissue displayed a significant 3 h shift (i.e., hippocampus, cerebellum) or no shift (i.e., septum, olfactory bulb). For each tissue, similar results were obtained for the core clock genes *Per1*, *Cry1*, and *Cry2*, with the rhythms in each likewise blunted in a nearly universal manner (Fig. 4c, Additional file 1: Figure S3 and Additional file 1: Table S3). Thus, long day lengths that dissociate the SCN network markedly reduce the amplitude of core clock gene rhythms in four distinct central brain structures.

**Fig. 5:**
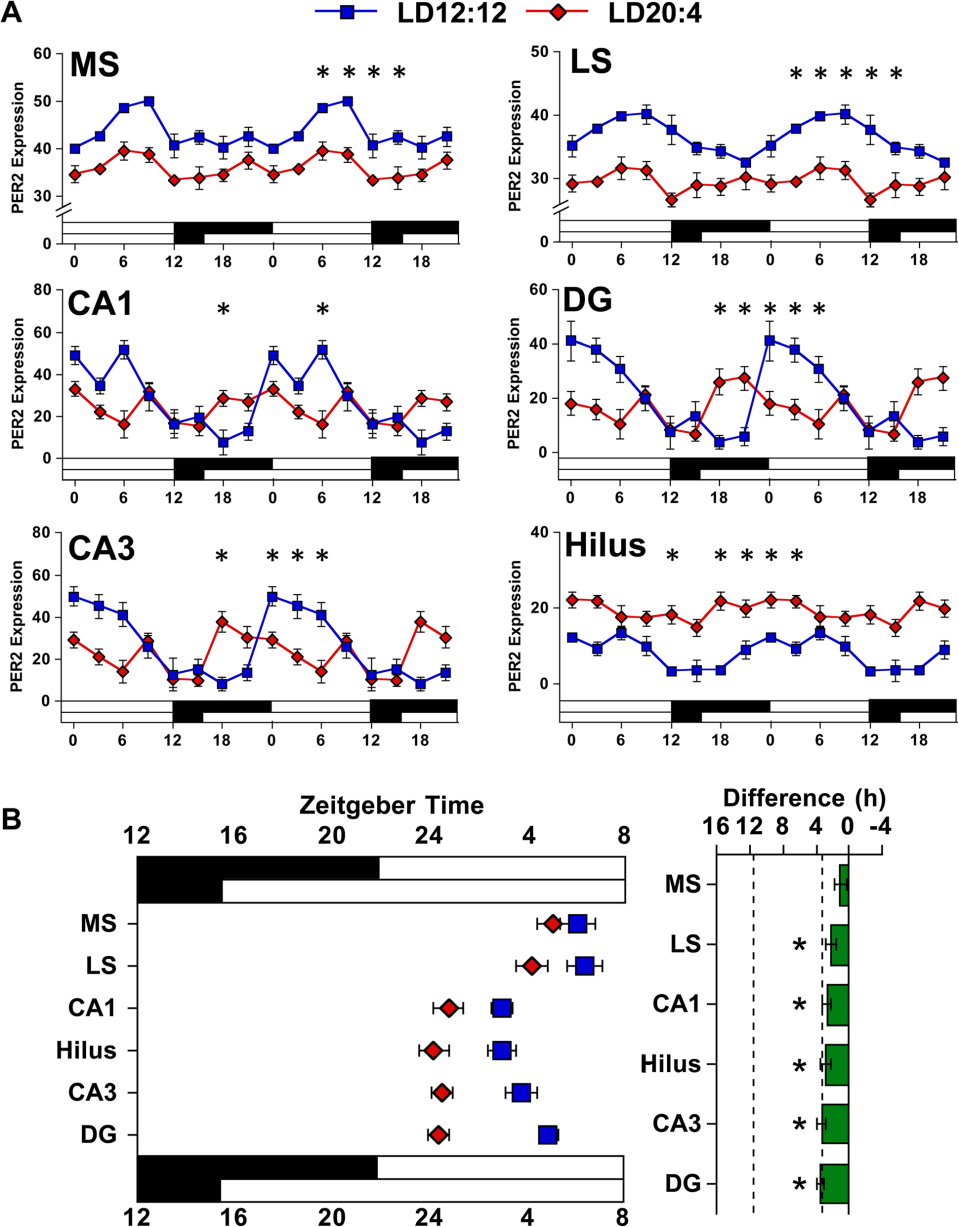
The phase and amplitude of PER2 rhythms in central tissues is influenced by photoperiod. **a** Double-plotted rhythms in PER2 expression were measured with immunohistochemistry for the subdivisions of the septum [medial septum (MS), lateral septum (LS)] and hippocampus (CA1, CA3, Dentate Gyrus, Hilus) under LD12:12 (blue symbols) and LD20:4 (red symbols). White and black bars on the abscissa represent lighting conditions. *n* = 4–6/time-point/photoperiod. * LD12:12 versus LD20:4, LS Means Contrasts, *P* <0.006. Cosinor analyses of PER2 rhythms are shown in Additional file 1: Table S4. **b** Summary plots of photoperiodic changes in the phase of central tissues. Tissues are ordered by the magnitude of the difference in peak time. Dashed vertical lines indicate the magnitude of the shift displayed by the SCN shell and SCN core *in vivo*, as detected with PER2 immunohistochemistry (c.f., Additional file 1: Table S4). * Significant phase difference, one sample *t*-test, *P* <0.05

Although highly consistent across tissues, the reduced amplitude could reflect that subregions of each tissue shifted in different directions. To test whether decreased *Per2* amplitude reflected differential responses at the microcircuit level, PER2 rhythms were analyzed in the hippocampus and septum using immunohistochemistry (Additional file 1: Figure S4, and Figure S5). Consistent with our *Per2* results, PER2 expression was rhythmic under LD12:12 in each tissue and subregion examined (Fig. 5, Additional file 1: Table S4). Overall, changes in the phase and amplitude of PER2 expression under LD20:4 were consistent with our *Per2* results for both the septum and hippocampus. Under LD20:4, both the lateral and medial subdivisions of the septum displayed an arrhythmic pattern of PER2 expression with a large decrease in amplitude (Fig. 5, Additional file 1: Table S4). In the hippocampus, LD20:4 advanced the time of peak PER2 expression by 3–6 h in each of the four subregions examined, and the amplitude of PER2 rhythms was decreased consistently, with the magnitude of this decrease dependent on subregion (Fig. 5, Additional file 1: Table S4). Thus, we observed that substructures within each tissue shifted in the same direction, shifted by a similar amount, and displayed a marked reduction in amplitude. Collectively, these data indicate that the reduction in amplitude under LD20:4 does not reflect differential responses at the microcircuit level, but instead a consistent decrease in amplitude that is evident across tissues and subregions at both the transcriptional and translational level.

### SCN dissociation diminishes shell output

**Fig. 6:**
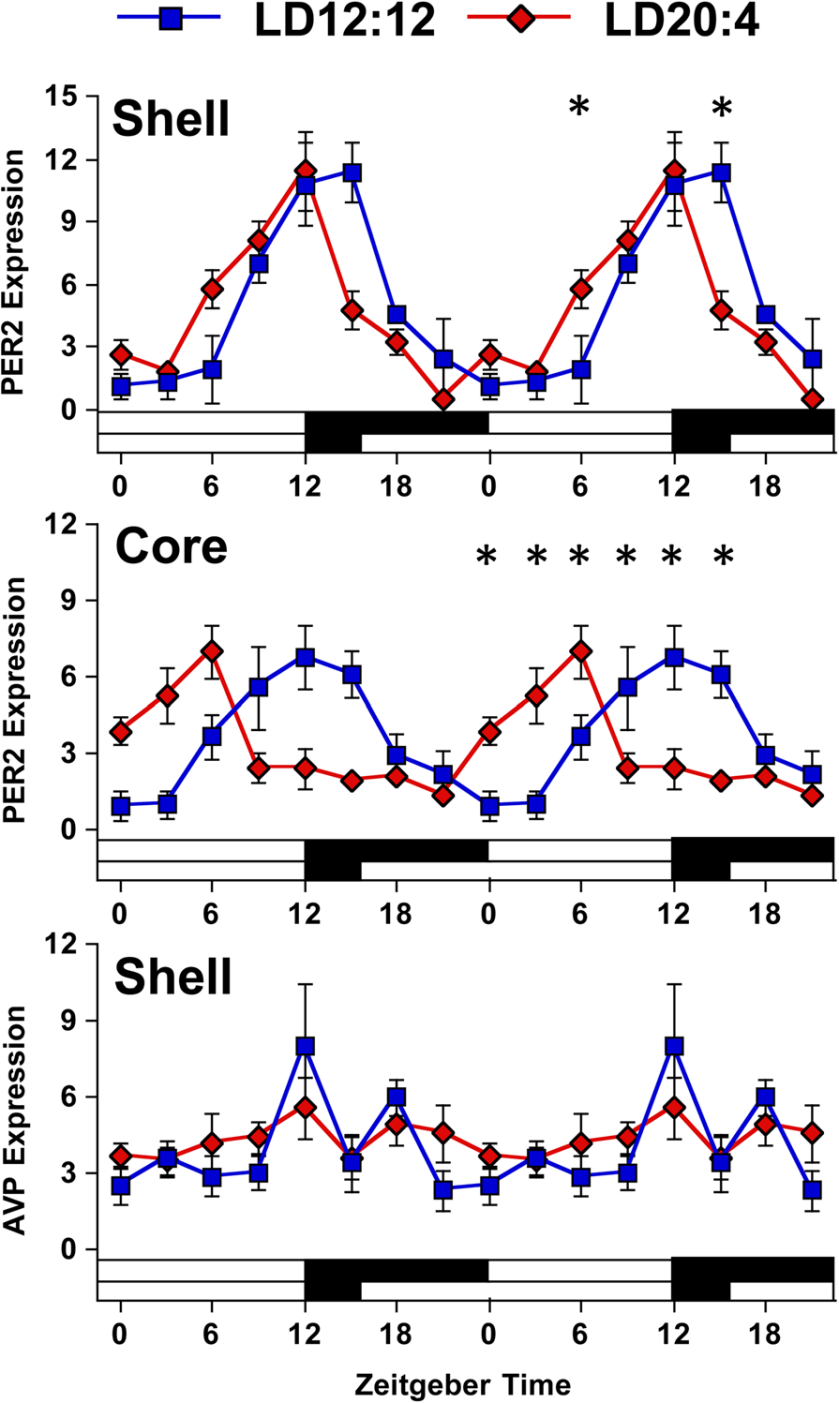
The amplitude of AVP expression in the SCN is decreased by long day photoperiods. Double-plotted rhythms of PER2 and AVP expression measured in the SCN with immunohistochemistry. White and black bars on the abscissa represent lighting conditions. *n* = 4–8/time-point/photoperiod. *LD12:12 versus LD20:4, LS Means Contrasts, *P* <0.006. Cosinor analyses of PER2 and AVP rhythms are in Additional file 1: Table S4

The marked reduction in the amplitude of rhythms in downstream tissues may indicate that there are functionally important outputs from both SCN compartments, with the SCN shell setting the phase of downstream tissues and the SCN core influencing amplitude. On the other hand, these results may reflect that the amplitude of rhythms in the SCN shell have been diminished by long day lengths, which would not require direct communication from the SCN core to downstream tissues. To distinguish between these two hypotheses, we tested whether LD20:4 decreased the amplitude of rhythms in the SCN shell using immunohistochemistry (Additional file 1: Figure S6, and Figure S7). Consistent with previous work [21] and the PER2::LUC imaging data described above, PER2 rhythms in the SCN shell and core were similarly phased under LD12:12 but adopted different peak times under LD20:4 (Fig. 6, Additional file 1: Table S4, see also [21]). Despite the change in phase relationship, LD20:4 did not decrease the amplitude of PER2 rhythms in either the SCN shell or SCN core (Fig. 6, Additional file 1: Table S4). To test changes in the amplitude of outputs from the SCN shell, we next analyzed rhythms in AVP protein expression (Additional file 1: Figure S7), which is a major output of the SCN shell [33]. Consistent with previous work, AVP expression in the SCN was rhythmic under LD12:12 (Fig. 6, Additional file 1: Table S4). In contrast, AVP expression was not rhythmic under LD20:4, with a 50 % reduction in amplitude of expression due to higher expression during daytime and lower expression during nighttime, relative to LD12:12 (Fig. 6, Additional file 1: Table S4). This suggests that outputs from the SCN shell are attenuated under long day photoperiods, with light-driven changes in the functional organization of the SCN producing a decrease in the amplitude of AVP rhythms. Given the import of this SCN shell output, this may provide a parsimonious explanation for diminished amplitude of clock gene/protein rhythms in downstream tissues.

## Discussion

The SCN is important for coordinating downstream tissues in the circadian system so that they are synchronized to one another and the environment. Herein, we have used *in vivo* lighting conditions to differentially shift the SCN shell and core to assess the functional role of region-specific outputs to peripheral and central tissues. Overall, our findings demonstrate that downstream tissues maintain phase synchrony with the SCN shell, but not the SCN core. Additionally, we found that light-driven reorganization of the SCN network produced a striking decrease in the amplitude of clock gene/protein rhythms in central brain tissues. These data support a model where the SCN shell coordinates phase at the system level, but yet the integrated function of the SCN network is necessary for maintaining high amplitude rhythms in downstream tissues. Further, this study reveals that photoperiod can modulate the amplitude of molecular rhythms in local clocks of non-SCN tissues, which is a new form of circadian plasticity that may contribute to seasonal changes in physiology and behavior.

It has been known for some time that the SCN sets the phase of downstream tissues and that both of its main compartments can influence the properties of overt rhythms [34]. Previous neuroanatomical work in different rodent species suggested that the SCN shell and core project to both common and distinct targets [5, 13, 17, 35], but the relevance of regional projections for downstream clock function remained unclear. The current study represents the most comprehensive attack on this issue to date by examining how SCN dissociation changes the molecular clock of downstream tissues. Nearly all of the tissues examined here maintained phase synchrony with the SCN shell and not the SCN core. Additionally, we find that the correspondence between the phase of the SCN shell and peripheral tissues was maintained after release into constant darkness, which suggests that light is not directly masking the phase of peripheral tissues. We further attempted to evaluate the potential role of light exposure and overt rhythms by investigating re-entrainment of locomotor and sleep rhythms under LD20:4 (Additional file 1: Figure S1). These two specific rhythms displayed small to negligible shifts, similar to three tissues that failed to display a significant phase shift under LD20:4 (i.e., the kidney, the spleen, and the olfactory bulb). Thus, the phase of these three specific tissues may be masked by light or influenced by overt rhythms that do not shift under LD20:4. However, the vast majority of tissues shift to a degree that suggests they receive time-of-day cues provided by the SCN shell. Although we have sampled a wide range of tissues and systems, not all peripheral and central tissues were examined, and thus it remains possible that the SCN core sets the phase of clocks not included here. This caveat aside, this study supports a model emphasizing the role of the SCN shell in transmitting time-of-day cues to the wider circadian system [33].

The mechanisms underlying the regulation of phase by the SCN shell may involve direct synaptic connections, release of humoral signals, and indirect control via other tissues or overt rhythms [3]. Herein, we have taken the first steps towards evaluating potential resetting signals from the SCN shell by examining photoperiodic changes in AVP expression, which is a major SCN output [33]. Because we find that AVP expression is arrhythmic under long days, this suggests that downstream tissues are not reset by AVP specifically, but by other signals produced by the SCN shell that remain rhythmic [36–38]. Although it is possible an arrhythmic AVP output could serve as a resetting signal in the presence of rhythmic responses in downstream tissues [39, 40], it is unlikely that all 17 tissues studied here are reset by one common signal given evidence that different target systems rely on different SCN signaling pathways, including from synaptic connections [41–43], humoral factors [44, 45], and indirect control via overt rhythms [29]. Furthermore, retrograde tract tracing studies conducted in the rat indicate that downstream tissues receive inputs from both AVP and non-AVP neurons in the SCN shell [46]. While the specific output mechanisms that set the phase of peripheral tissues remain unclear, the current results serve to focus attention on signals provided by SCN neurons in the shell compartment.

Unexpectedly, we found that exposure to long day photoperiods also produced a striking decrease in the amplitude of clock gene and protein expression in several distinct brain structures, which was sufficient to abolish the rhythm in some of these tissues. Given the 3 h temporal resolution of the current analyses and the consistency of these results, it is highly unlikely that the decreased amplitude is due to a “missed” peak or trough. Additionally, the brain tissues examined here do not receive retinal innervation [47], which suggests that the decrease in amplitude is not a direct response to longer light exposure. Further, previous work suggests that rhythms in *Per2*/PER2 expression in peripheral tissues are specifically regulated by systemic cues controlled by the SCN [29, 48, 49]. We find that reduced amplitude was not attributable to desynchrony at the microcircuit level, although it remains possible that cells within each region were desynchronized under long day lengths. Future work employing bioluminescence imaging of these brain structures may be able to resolve this, although use of this technology may be difficult given that neural tissues can be reset by the dissection procedure itself [31, 32].

Consistent with the decreased amplitude of core clock gene/protein expression in central tissues, we find that the daily rhythm in AVP expression in the SCN shell was diminished under long day lengths. Because AVP serves as both an output itself and regulator of other SCN outputs, such as prokineticin2 [37], this may indicate that the overall strength of outputs from the SCN shell are compromised under long day lengths. This has important implications because it indicates that the amplitude of overt rhythms may be modulated by targeting the SCN shell specifically. Further, the correspondence between the loss of AVP rhythms under LD20:4 and the reduced amplitude of core clock gene/protein rhythms in downstream tissues may reflect an important role for AVP in modulating the amplitude of the molecular clock in downstream tissues. Consistent with this, previous work indicates that AVP can regulate the amplitude of overt rhythms, including the expression of corticosterone, sleep, and melatonin [37, 50–52]. Considering these changes occur in a variety of overt rhythms, it remains possible that photoperiodic modulation of amplitude is more widespread than detected here. For example, changes in the amplitude of PER2::LUC rhythms may have been occluded by the effects of tissue collection, although dissection-induced effects on amplitude were not evident in 12/13 of the tissues retained in our analyses.

Thus, amplitude suppression in central structures is associated with reduced amplitude of AVP expression in the SCN, which prompts the question: What might cause the decrease in this output signal? We propose that this reflects the light-driven change in the relative phase of the SCN core, which during an integrated state provides cues to amplify rhythmic outputs from the SCN shell [53]. Thus, according to this model, the internal misalignment of the SCN network is the ultimate cause of reduced amplitude in downstream clocks. Consistent with this hypothesis, recent work using a forced desynchrony model in rats demonstrates that SCN dissociation decreases the daily peak in luteinizing hormone and corticosterone secretion [17, 19] and alters the waveform of daily melatonin release [20]. Based on these previous results and our current data, we propose that light-driven SCN dissociation produces a widespread reduction in amplitude that is not specific to hormonal rhythms. Our current results suggest that this reduction in amplitude reflects a decrease in the strength of SCN shell outputs caused by the change in the functional organization of the SCN network. Within the context of this model, the SCN core need not directly innervate downstream tissues, although it remains possible that the SCN core provides cues that directly modulate the amplitude of downstream tissues. Because it is clear that SCN core neurons do indeed form connections with other structures in the brain [5, 13, 35, 54], further work is warranted to investigate the functional relevance of these outputs. Regardless, the current observation that lighting conditions modulate amplitude in downstream tissues reveals a new form of molecular plasticity that may serve to encode day length and contribute to seasonal changes in physiology and behavior.

## Conclusions

**Fig. 7:**
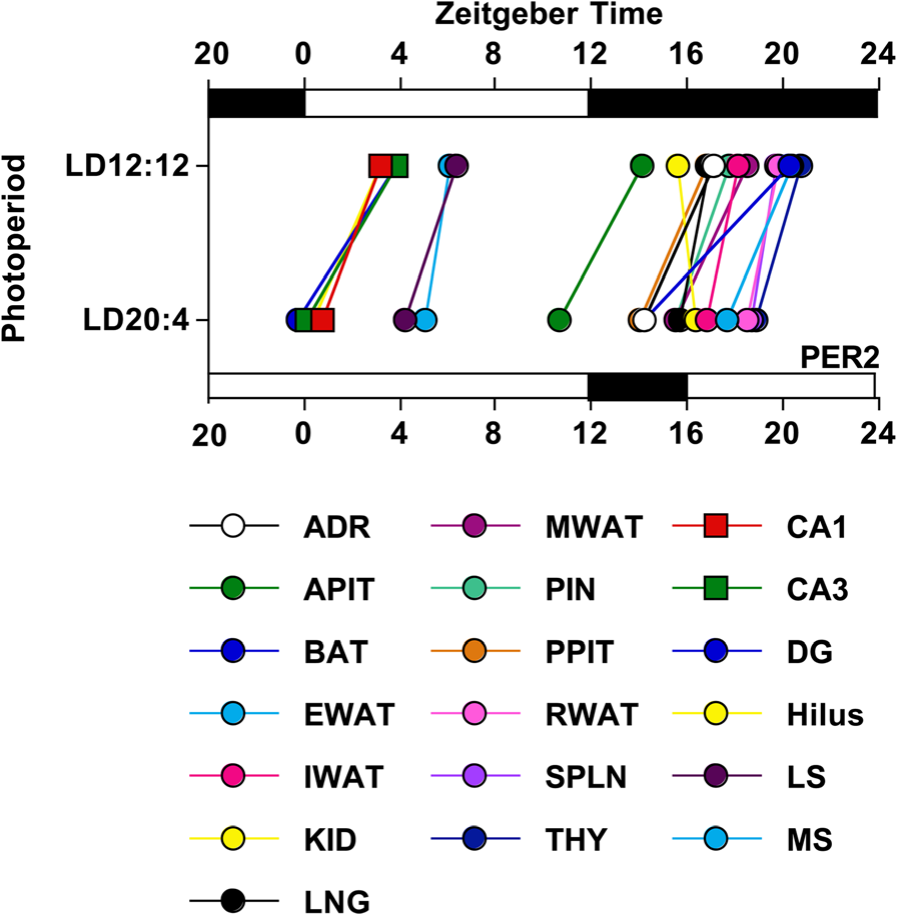
Summary of photoperiodic modulation of phase relationships among tissues in the circadian system. Phase relationships among tissues indicated by the ZT peak time of PER2 expression. Lines connect the same tissue collected from mice entrained to LD12:12 (top) versus LD20:4 (bottom). ZT peak time was determined using PER2::LUC for the adrenal gland (ADR), anterior pituitary gland (APIT), brown adipose tissue (BAT), cornea (CORN), esophagus (ESO), epididymal white adipose tissue (EWAT), inguinal white adipose tissue (IWAT), kidney (KID), liver (LIV), lung (LNG), mesenteric white adipose tissue (MWAT), pineal gland (PIN), posterior pituitary gland (PPIT), retina (RET), retinal pigmented epithelium (RPE), retroperitoneal white adipose tissue (RWAT), spleen (SPLN), and thymus (THY). ZT peak time was determined using PER2 immunohistochemistry for regions of the hippocampus (CA1, CA3, DG, Hilus) and septum (LS, MS)

The current study suggests that there is a general reliance on the SCN shell for setting the phase of downstream tissues, which may be an adaptive design feature critical for maintaining system-level temporal organization in a changing environment. Using the current results as an indication, the specialization of the SCN shell in providing time-of-day cues would appear to conserve system-level phase relationships during seasonal changes in day length (Fig. 7). A division of labor among SCN compartments may be of particular importance given findings that the spatiotemporal organization of the SCN network can be markedly altered by both acute and chronic changes in environmental conditions (present results, [21, 55]). Overall, downstream clocks were highly consistent in the direction of phase shifts displayed under long day lengths; however, differences in the magnitude of these phase shifts did alter some phase relationships across and within tissues (Fig. 7). In contrast to the modest changes in phase relationships observed here, an abrupt shift in the light:dark cycle (i.e., simulated jetlag) produces large changes in oscillator phase relationships at the system-level due to differences in the rate and direction of resetting across tissues [55–57]. Given recent work linking depression to altered phase relationships at the system and molecular level [58], a critical question for future study is whether the photoperiodic modulation observed in the current study alters behavioral and physiological indices known to vary with season and circadian disruption [4].

## Methods

### Mice

Homozygous PER2::LUC knockin mice [24], backcrossed onto a C57BL/6 background, were bred and raised under a 24 h light:dark (LD) cycle with 12 h of light and 12 h of darkness (LD12:12, lights off: 1800 EST). Throughout life, ambient temperature was maintained at 22 ± 2 °C, and animals had *ad libitum* access to water and food (Purina Rodent Chow #5001, St Louis, MO). At weaning, animals were group-housed in cages without running wheels. At 6–10 weeks of age, male PER2::LUC mice were transferred to individual wheel-running cages housed within light- and temperature-controlled secondary enclosures. For at least 12 weeks, mice were either maintained under LD12:12 or re-entrained to a long day photoperiod with 20 h of light (LD20:4, lights off: 2200 EST). All procedures were conducted according to the NIH Guide for the Care and Use of Animals and were approved by the Institutional Animal Care and Use Committees at the Morehouse School of Medicine and Marquette University.

### PER2::LUC tissue culture

Male PER2::LUC mice were sacrificed with CO_2_ asphyxiation and cervical dislocation prior to tissue collection at specific times relative to lights-off (defined as Zeitgeber Time 12, ZT12). Brains were removed, bathed in chilled Hank’s solution, and sectioned in the coronal plane with a motorized vibratome. SCN slices (150 μm) were imaged using a Stanford Photonics MEGA-10Z cooled intensified CCD camera housed in a light-tight environmental chamber set at 36.5 °C, as described previously [21]. Other tissues were excised, placed in chilled Hank’s solution, and trimmed by hand with a scalpel, as described previously [55]. A wide variety of tissues was selected to represent a cross-section of multiple physiological systems, with assessment of adrenal gland, anterior pituitary gland, posterior pituitary gland, pineal gland, lung, spleen, thymus, esophagus, liver, kidney, retina, retinal pigmented epithelium, cornea, brown adipose tissue, epididymal white adipose tissue, inguinal white adipose tissue, mesenteric white adipose tissue, and retroperitoneal white adipose tissue. All tissues were cultured on a membrane with 1.2 mL of air-buffered medium containing 0.1 mM beetle luciferin (Gold Biotechnologies). Bioluminescence rhythms from peripheral tissues were measured with a luminometer (Actimetrics Inc., Evanston, IL) housed inside a light-tight incubator set at 36 °C. For bioluminescence imaging analyses, SCN shell and core regions were identified visually based on spatial location (Fig. 1), as in [21]. PER2::LUC time series were corrected for the ZT12 start time of the recording and de-trended by subtracting the 24 h running average from the raw data. PER2::LUC time series were analyzed with Lumicycle analysis software (Actimetrics) by fitting a damped sine wave to the first three cycles *in vitro*, starting with the time of the first trough *in vitro*. Peak time was recorded as the first peak within this recording interval and expressed relative to projected ZT12 *in vitro*, as in previous studies [55–57]. This method has been established for inferring *in vivo* phase, but similar results were obtained when the time of peak PER2::LUC expression *in vitro* was directly compared across photoperiodic conditions.

### qRT-PCR

Male PER2::LUC mice from each photoperiod were euthanized with CO_2_ asphyxiation and cervical dislocation at time-points spanning the circadian cycle (*n* = 3/time-point/photoperiod). Brains were immediately post fixed in 4 % paraformaldehyde for 6 h, then cryoprotected in 20 % sucrose solution for 3–5 days. A variety of central brain structures were retained to represent a cross-section of different behavioral systems, with assessment of the cerebellum, hippocampus, olfactory bulb, and septum. Brains were blocked and one lobe of the olfactory bulb and cerebellum was retained before brains were sectioned on a cryostat (100 μm). Hippocampus and septum were excised by hand with a scalpel from three consecutive slices. Tissue samples were homogenized and digested overnight at 55 °C in a lysis buffer supplemented with 50 μL of proteinase K (20 mg/mL). Total RNA was purified with TRIzol, mixed with chloroform, and then centrifuged for 15 min. RNA was precipitated with 70 % isopropanol and centrifuged for 15 min. Resulting pellets were washed with 75 % ethanol and centrifuged for 15 min. Pellets were then dried and re-suspended with 10 μL RNAase DNAase free water. RNA concentration and purity was quantified with 260/280 nm absorbance ratios measured with a Nanodrop 1000 spectrophotometer (Thermo Scientific, Wilmington, DE). First strand cDNA synthesis was achieved with reverse transcription using a high-capacity cDNA reverse transcription kit (Applied Biosystems, Cat#4368813). RNA and cDNA was stored at −20 °C prior to processing. qRT-PCR assays were performed with a BioRad or Applied Biosystem thermocycler using Sso Advanced SYBR Green Supermix (Cat#172-5261). The thermal cycling program was set for 95 °C for 15 s, 60 °C for 30 s, and 72 °C for 45 s for 40 cycles, followed by melt curve analysis using a 0.5 °C 5 s stepwise gradient from 60 °C to 95 °C. Sequences of the primers used are listed in Additional file 1: Table S5. *P0* was selected as the reference gene based on its stable expression across the circadian cycle relative to four other commonly used reference genes (*βactin*, *18S*, *cyclophilin*, *gapdh*). Cycle thresholds were analyzed with the ΔΔCT method by normalizing to the average of gene expression under LD12:12 collapsed across time-points.

### Immunohistochemistry

Brains were collected as described above and sectioned on a cryostat (40 μm). Slices containing SCN and non-SCN structures were retained and stored in cryoprotectant at −20 °C. Free-floating slices were washed in 0.1 M phosphate-buffered solution (PBS) and then incubated for 48 h at 4 °C with primary antibodies (Non-SCN: PER2, 1:1 K, Millipore Cat#AB2202, SCN: PER2, 1:2 K and AVP, 1:4 K, Bachem Cat#T-5048). Slices were then washed in PBS before 2 h incubation at room temperature with secondary antibodies (Jackson ImmunoResearch, Alexa Flour 488 Cat#711-545-152, Alexa Flour 594 Cat#706-585-148; both at 1:500). Slices were then washed with PBS and mounted using Promount anti-fade gold reagent (Cat#P36935). Fluorescence images were obtained with a Zeiss LMS 700 confocal microscope using sequential scanning and identical settings for all samples. Z stack images were compressed and analyzed with ImageJ. Specific signal intensity was quantified by subtracting background intensity values. For SCN analyses, PER2 expression in SCN shell and core regions was quantified by identifying neurons with and without AVP immunoreactivity (ir), respectively (also see [21]). PER2-ir and AVP-ir cells were identified with ImageJ software using the Analyze Particles method. Briefly, the ten consecutive z-stack images were summed, the summed image was duplicated, converted to 8-bit grayscale, and then thresholded to produce a binary image (Additional file 1: Figure S6, and S7). After watershed separation, cell-like regions of interest were identified in each image using size and circularity criteria to distinguish cells from fibers and non-specific staining. Average signal intensity value for the cell-like regions of interest was then calculated by referencing the original, non-thresholded image. For non-SCN structures with moderate to low cell density (Medial Septum, Lateral Septum, Hilus), average signal intensity was calculated for PER2-ir neurons using the Analyze Particles method. For non-SCN structures with higher cell density (CA1, CA3, dentate gyrus), PER2-ir was calculated using the average intensity values of five larger regions of interest manually positioned onto the relevant structure for each image.

### Behavioral analyses

Wheel running rhythms were monitored and analyzed with the Clocklab data collection and analysis system (Actimetrics) as previously described [21]. Briefly, activity onset was identified as the first bin above a threshold of five counts that was preceded by at least 2 h of inactivity and followed by at least 1 h of activity likewise above threshold. Wheel-running revolutions were calculated during the light and dark phases for the last week of entrainment using the average activity profile for each mouse. Sleep data were collected and analyzed as previously outlined [59]. Briefly, electroencephalogram (EEG) and electromyography (EMG) electrodes (frontal electrode: 1.5 mm anterior to bregma, 1.5 mm lateral to central suture; interparietal electrodes: 2.5 mm posterior to bregma, ±1.5 mm lateral to central suture) were implanted in anesthetized mice using a prefabricated head mount (integrated 2 × 3 pin grid array, Pinnacle Technologies, Lawrence, KS). EMG activity was monitored using stainless-steel Teflon-coated wires that were inserted bilaterally into the nuchal muscle. Two weeks after surgery, mice were moved to the sleep-recording chamber and connected to a lightweight tether attached to a low-resistance commutator mounted over the cage (Pinnacle Technologies, Lawrence, KS). EEG and EMG recording began following one-week acclimation to the tether and recording chamber. Data were collected with Sirenia Acquisition software (Pinnacle Technologies). EEG signals were low-pass filtered with a 40-Hz cutoff and collected continuously at a sampling rate of 400 Hz. All recordings were scored offline in 10 s epochs. All waveforms were classified by a trained observer using both EEG leads and EMG into the following states: Wake (low voltage, high frequency EEG; high amplitude EMG), non-rapid eye movement sleep (high voltage, mixed frequency EEG; low amplitude EMG), or rapid eye movement sleep (low voltage EEG with a predominance of theta activity (6–10 Hz); very low amplitude EMG). EEG epochs determined to have an artifact (interference caused by scratching, movement, eating, or drinking) were excluded from analyses.

### Statistical analyses

Statistical analyses were performed with JMP software (SAS Institute, Cary, NC) or CircWave software [60]. Data are represented in figures and tables as mean ± SEM.

## Additional file

Additional file 1: Table S1. PER2::LUC rhythms from non-SCN tissues collected at two different times of day. Table S2. Photoperiodic changes in PER2::LUC rhythms of non-SCN tissues. Table S3. Photoperiodic changes in clock gene rhythms of non-SCN tissues. Table S4. Photoperiodic changes in protein rhythms of SCN and non-SCN tissues. Table S5. Primers used for qRT-PCR. Figure S1. Re-entrainment of locomotor activity and sleep rhythms under LD20:4. Figure S2. The vast majority of peripheral tissues were not reset by culture. Figure S3. The phase and amplitude of core clock gene rhythms in central tissues is influenced by photoperiod. Figure S4. Representative, background-subtracted images representing PER2 expression in the hippocampus of mice held under LD12:12 and LD20:4 measured with immunohistochemistry. Figure S5. Representative, background-subtracted images representing PER2 expression in the septum of mice held under LD12:12 and LD20:4 measured with immunohistochemistry. Figure S6. Representative, background-subtracted images representing PER2 expression in the SCN of mice held under LD12:12 and LD20:4 measured with immunohistochemistry. Figure S7. Representative, background-subtracted images representing AVP expression in the SCN of mice held under LD12:12 and LD20:4 measured with immunohistochemistry.

## Abbreviations

AVP: Arginine vasopressin
Cry1: Cryptochrome1
Cry2: Cryptochrome2
EEG: Electroencephalogram
EMG: Electromyography
LD: Light:dark
LD12:12: Standard lighting condition with 12 h of light
LD20:4: Long day with 20 h of light
LL: Constant light
Per1: Period1
Per2: Period2
PER2::LUC: PERIOD2::LUCIFERASE
SCN: Suprachiasmatic nucleus
VIP: Vasoactive intestinal polypeptide

## References

[1] Mohawk JA, Green CB, Takahashi JS (2012). Central and peripheral circadian clocks in mammals. Annu Rev Neurosci.

[2] Ko CH, Takahashi JS (2006). Molecular components of the mammalian circadian clock. Hum Mol Genet.

[3] Dibner C, Schibler U, Albrecht U (2010). The mammalian circadian timing system: Organization and coordination of central and peripheral clocks. Annu Rev Physiol.

[4] Evans JA, Davidson AJ (2013). Health consequences of circadian disruption in humans and animal models. Prog Mol Biol Transl Sci.

[5] Abrahamson EE, Moore RY (2001). Suprachiasmatic nucleus in the mouse: retinal innervation, intrinsic organization and efferent projections. Brain Res.

[6] Antle MC, Foley DK, Foley NC, Silver R (2003). Gates and oscillators: a network model of the brain clock. J Biol Rhythms.

[7] Nagano M, Adachi A, Nakahama K, Nakamura T, Tamada M, Meyer-Bernstein E (2003). An abrupt shift in the day/night cycle causes desynchrony in the mammalian circadian center. J Neurosci.

[8] Sumova A, Illnerova H (2005). Effect of photic stimuli disturbing overt circadian rhythms on the dorsomedial and ventrolateral SCN rhythmicity. Brain Res.

[9] Nakamura W, Yamazaki S, Takasu NN, Mishima K, Block GD (2005). Differential response of Period 1 expression within the suprachiasmatic nucleus. J Neurosci.

[10] Albus H, Vansteensel MJ, Michel S, Block GD, Meijer JH (2005). A GABAergic mechanism is necessary for coupling dissociable ventral and dorsal regional oscillators within the circadian clock. Curr Biol.

[11] Morin LP (2007). SCN organization reconsidered. J Biol Rhythms.

[12] Kriegsfeld LJ, Leak RK, Yackulic CB, LeSauter J, Silver R (2004). Organization of suprachiasmatic nucleus projections in Syrian hamsters (*Mesocricetus auratus*): an anterograde and retrograde analysis. J Comp Neurol.

[13] Yan L, Foley NC, Bobula JM, Kriegsfeld LJ, Silver R (2005). Two antiphase oscillations occur in each suprachiasmatic nucleus of behaviorally split hamsters. J Neurosci.

[14] de la Iglesia HO, Meyer J, Carpino A, Schwartz WJ (2000). Antiphase oscillation of the left and right suprachiasmatic nuclei. Science.

[15] Ohta H, Yamazaki S, McMahon DG (2005). Constant light desynchronizes mammalian clock neurons. Nat Neurosci.

[16] Butler MP, Rainbow MN, Rodriguez E, Lyon SM, Silver R (2012). Twelve-hour days in the brain and behavior of split hamsters. Eur J Neurosci.

[17] Smarr BL, Morris E, de la Iglesia HO (2012). The dorsomedial suprachiasmatic nucleus times circadian expression of *Kiss1* and the luteinizing hormone surge. Endocrinology.

[18] Lee ML, Swanson BE, de la Iglesia HO (2009). Circadian timing of REM sleep is coupled to an oscillator within the dorsomedial suprachiasmatic nucleus. Curr Biol.

[19] Wotus C, Lilley TR, Neal AS, Suleiman NL, Schmuck SC, Smarr BL (2013). Forced desynchrony reveals independent contributions of suprachiasmatic oscillators to the daily plasma corticosterone rhythm in male rats. PLoS One.

[20] Schwartz MD, Wotus C, Liu T, Friesen WO, Borjigin J, Oda GA (2009). Dissociation of circadian and light inhibition of melatonin release through forced desynchronization in the rat. Proc Natl Acad Sci U S A.

[21] Evans JA, Leise TL, Castanon-Cervantes O, Davidson AJ (2013). Dynamic interactions mediated by nonredundant signaling mechanisms couple circadian clock neurons. Neuron.

[22] Inagaki N, Honma S, Ono D, Tanahashi Y, Honma K (2007). Separate oscillating cell groups in mouse suprachiasmatic nucleus couple photoperiodically to the onset and end of daily activity. Proc Natl Acad Sci U S A.

[23] Naito E, Watanabe T, Tei H, Yoshimura T, Ebihara S (2008). Reorganization of the suprachiasmatic nucleus coding for day length. J Biol Rhythms.

[24] Yoo SH, Yamazaki S, Lowrey PL, Shimomura K, Ko CH, Buhr ED (2004). PERIOD2::LUCIFERASE real-time reporting of circadian dynamics reveals persistent circadian oscillations in mouse peripheral tissues. Proc Natl Acad Sci U S A.

[25] Yoshikawa T, Yamazaki S, Menaker M (2005). Effects of preparation time on phase of cultured tissues reveal complexity of circadian organization. J Biol Rhythms.

[26] Ishida A, Mutoh T, Ueyama T, Bando H, Masubuchi S, Nakahara D (2005). Light activates the adrenal gland: timing of gene expression and glucocorticoid release. Cell Metab.

[27] Redlin U, Mrosovsky N (1999). Masking by light in hamsters with SCN lesions. J Comp Physiol A.

[28] Husse J, Leliavski A, Tsang AH, Oster H, Eichele G (2014). The light–dark cycle controls peripheral rhythmicity in mice with a genetically ablated suprachiasmatic nucleus clock. FASEB J.

[29] Son GH, Chung S, Choe HK, Kim HD, Baik SM, Lee H (2008). Adrenal peripheral clock controls the autonomous circadian rhythm of glucocorticoid by causing rhythmic steroid production. Proc Natl Acad Sci U S A.

[30] Kiessling S, Sollars PJ, Pickard GE (2014). Light stimulates the mouse adrenal through a retinohypothalamic pathway independent of an effect on the clock in the suprachiasmatic nucleus. PLoS One.

[31] Guilding C, Hughes AT, Brown TM, Namvar S, Piggins HD (2009). A riot of rhythms: neuronal and glial circadian oscillators in the mediobasal hypothalamus. Mol Brain.

[32] Guilding C, Hughes AT, Piggins HD (2010). Circadian oscillators in the epithalamus. Neuroscience.

[33] Kalsbeek A, Fliers E, Hofman MA, Swaab DF, Buijs RM (2010). Vasopressin and the output of the hypothalamic biological clock. J Neuroendocrinol.

[34] Weaver DR (1998). The suprachiasmatic nucleus: a 25-year retrospective. J Biol Rhythms.

[35] Leak RK, Moore RY (2001). Topographic organization of suprachiasmatic nucleus projection neurons. J Comp Neurol.

[36] Zhou QY, Cheng MY (2005). Prokineticin 2 and circadian clock output. FEBS J.

[37] Li JD, Burton KJ, Zhang C, Hu SB, Zhou QY (2009). Vasopressin receptor V1a regulates circadian rhythms of locomotor activity and expression of clock-controlled genes in the suprachiasmatic nuclei. Am J Physiol Regul Integr Comp Physiol.

[38] Kramer A, Yang FC, Snodgrass P, Li X, Scammell TE, Davis FC (2001). Regulation of daily locomotor activity and sleep by hypothalamic EGF receptor signaling. Science.

[39] Yoder JM, Brandeland M, Engeland WC (2014). Phase-dependent resetting of the adrenal clock by ACTH *in vitro*. Am J Physiol Regul Integr Comp Physiol.

[40] Wu C, Sui G, Archer SN, Sassone-Corsi P, Aitken K, Bagli D (2014). Local receptors as novel regulators for peripheral clock expression. FASEB J.

[41] de la Iglesia HO, Meyer J, Schwartz WJ (2003). Lateralization of circadian pacemaker output: Activation of left- and right-sided luteinizing hormone-releasing hormone neurons involves a neural rather than a humoral pathway. J Neurosci.

[42] Meyer-Bernstein EL, Jetton AE, Matsumoto SI, Markuns JF, Lehman MN, Bittman EL (1999). Effects of suprachiasmatic transplants on circadian rhythms of neuroendocrine function in golden hamsters. Endocrinology.

[43] Teclemariam-Mesbah R, Ter Horst GJ, Postema F, Wortel J, Buijs RM (1999). Anatomical demonstration of the suprachiasmatic nucleus-pineal pathway. J Comp Neurol.

[44] Lehman MN, Silver R, Gladstone WR, Kahn RM, Gibson M, Bittman EL (1987). Circadian rhythmicity restored by neural transplant. Immunocytochemical characterization of the graft and its integration with the host brain. J Neurosci.

[45] Guo H, Brewer JM, Champhekar A, Harris RB, Bittman EL (2005). Differential control of peripheral circadian rhythms by suprachiasmatic-dependent neural signals. Proc Natl Acad Sci U S A.

[46] Ueyama T, Krout KE, Nguyen XV, Karpitskiy V, Kollert A, Mettenleiter TC (1999). Suprachiasmatic nucleus: a central autonomic clock. Nat Neurosci.

[47] Hattar S, Kumar M, Park A, Tong P, Tung J, Yau KW (2006). Central projections of melanopsin-expressing retinal ganglion cells in the mouse. J Comp Neurol.

[48] Lamia KA, Storch KF, Weitz CJ (2008). Physiological significance of a peripheral tissue circadian clock. Proc Natl Acad Sci U S A.

[49] Kornmann B, Schaad O, Bujard H, Takahashi JS, Schibler U (2007). System-driven and oscillator-dependent circadian transcription in mice with a conditionally active liver clock. PLoS Biol.

[50] Kalsbeek A, van der Vliet J, Buijs RM (1996). Decrease of endogenous vasopressin release necessary for expression of the circadian rise in plasma corticosterone: a reverse microdialysis study. J Neuroendocrinol.

[51] Brown MH, Nunez AA (1989). Vasopressin-deficient rats show a reduced amplitude of the circadian sleep rhythm. Physiol Behav.

[52] Schroder H, Stehle J, Henschel M (1988). Twenty-four-hour pineal melatonin synthesis in the vasopressin-deficient Brattleboro rat. Brain Res.

[53] Aton SJ, Colwell CS, Harmar AJ, Waschek J, Herzog ED (2005). Vasoactive intestinal polypeptide mediates circadian rhythmicity and synchrony in mammalian clock neurons. Nat Neurosci.

[54] van der Beek EM, Wiegant VM, van der Donk HA, van den Hurk R, Buijs RM (1993). Lesions of the suprachiasmatic nucleus indicate the presence of a direct vasoactive intestinal polypeptide-containing projection to gonadotrophin-releasing hormone neurons in the female rat. J Neuroendocrinol.

[55] Sellix MT, Evans JA, Leise TL, Castanon-Cervantes O, Hill DD, DeLisser P (2012). Aging differentially affects the re-entrainment response of central and peripheral circadian oscillators. J Neurosci.

[56] Davidson AJ, Castanon-Cervantes O, Leise TL, Molyneux PC, Harrington ME (2009). Visualizing jet lag in the mouse suprachiasmatic nucleus and peripheral circadian timing system. Eur J Neurosci.

[57] Yamazaki S, Numano R, Abe M, Hida A, Takahashi R, Ueda M (2000). Resetting central and peripheral circadian oscillators in transgenic rats. Science.

[58] Li JZ, Bunney BG, Meng F, Hagenauer MH, Walsh DM, Vawter MP (2013). Circadian patterns of gene expression in the human brain and disruption in major depressive disorder. Proc Natl Acad Sci U S A.

[59] Ehlen JC, Jefferson F, Brager AJ, Benveniste M, Paul KN (2013). Period-amplitude analysis reveals wake-dependent changes in the electroencephalogram during sleep deprivation. Sleep.

[60] Oster H, Damerow S, Hut RA, Eichele G (2006). Transcriptional profiling in the adrenal gland reveals circadian regulation of hormone biosynthesis genes and nucleosome assembly genes. J Biol Rhythms.

