# Supplementary material for: Shell neurons of the master circadian clock coordinate the phase of tissue clocks throughout the brain and body
Source: BMC Biol. 2015 Jun 23;13:43. doi: 10.1186/s12915-015-0157-x (PMC4489020; doi:10.1186/s12915-015-0157-x)
Supplement: Additional file 1: Table S1. — PER2::LUC rhythms from non-SCN tissues collected at two different times of day. Table S2. Photoperiodic changes in PER2::LUC rhythms of non-SCN tissues. Table S3. Photoperiodic changes in clock gene rhythms of non-SCN tissues. Table S4. Photoperiodic changes in protein rhythms of SCN and non-SCN tissues. Table S5. Primers used for qRT-PCR. Figure S1. Re-entrainment of locomotor activity and sleep rhythms under LD20:4. Figure S2. The vast majority of peripheral tissues were not reset by culture. Figure S3. The phase and amplitude of core clock gene rhythms in central tissues is influenced by photoperiod. Figure S4. Representative, background-subtracted images representing PER2 expression in the hippocampus of mice held under LD12:12 and LD20:4 measured with immunohistochemistry. Figure S5. Representative, background-subtracted images representing PER2 expression in the septum of mice held under LD12:12 and LD20:4 measured with immunohistochemistry. Figure S6. Representative, background-subtracted images representing PER2 expression in the SCN of mice held under LD12:12 and LD20:4 measured with immunohistochemistry. Figure S7. Representative, background-subtracted images representing AVP expression in the SCN of mice held under LD12:12 and LD20:4 measured with immunohistochemistry. [file 12915_2015_157_MOESM1_ESM.pdf]

Additional Files

Supplementary Tables

Table S1. PER2::LUC rhythms from non-SCN tissues collected at two different times of day.

Table S2. Photoperiodic changes in PER2::LUC rhythms of non-SCN tissues.

Table S3. Photoperiodic changes in clock gene rhythms of non-SCN tissues.

Table S4. Photoperiodic changes in protein rhythms of SCN and non-SCN tissues.

Table S5. Primers used for qRT-PCR.

Supplementary Figure Legends

Figure S1. Re-entrainment of locomotor activity and sleep rhythms under LD20:4. A) Representative double-plotted actograms depicting wheel-running rhythms of individual PER2::LUC mice entrained to LD12:12 or LD20:4. Light and dark phases of the photocycle are represented by the bar above each actogram and internal shading. B) Daily rhythms in total sleep, NREM sleep, and REM sleep under LD12:12 and LD20:4. n = 2-3/photoperiod. C) Summary plots of photoperiod-induced changes in the phase of locomotor and sleep rhythms, using center of gravity calculated by cosinor analyses (CircWave). Dashed vertical lines indicate the magnitude of the shift displayed by the SCN shell and SCN core. * Significant phase shift different from 0 h, one sample t test, $p < 0.05$. D) Photoperiod-induced changes in locomotor activity levels and amount of sleep. * Different from LD12:12, two sample t test, $p < 0.05$.

Figure S2. The vast majority of peripheral tissues were not reset by culture. A) Time of peak bioluminescence (± SEM) on the first cycle *in vitro* displayed by peripheral tissues collected either 2 h after lights-on (Zeitgeber Time 2, ZT2, open symbols) or 2 h before lights-off (ZT10,

filled symbols) from PER2::LUC mice housed under LD12:12. White and black bars on the upper and lower abscissa represent lighting conditions, with superimposed symbols representing the time of tissue culture at ZT2 or ZT10. n = 4-9/tissue/timepoint. B) Difference in the PER2::LUC peak time (± SEM) between cultures collected at ZT2 and ZT10. ADR: Adrenal gland, APIT: Anterior pituitary gland, BAT: Brown adipose tissue, CORN: Cornea, ESO: Esophagus, EWAT: Epididymal white adipose tissue, IWAT: Inguinal white adipose tissue, KID: Kidney, LIV: Liver, LNG: Lung, MWAT: Mesenteric white adipose tissue, PIN: Pineal gland, PPIT: posterior pituitary gland, RET: Retina, RPE: Retinal pigmented epithelium, RWAT: Retroperitoneal white adipose tissue, SPLN: Spleen, THY: Thymus. Tissues are ordered by the magnitude of difference in peak times. * Significant phase difference, one sample t test, $p < 0.05$. Rhythmic parameters and group comparisons for this dataset are summarized in Table S1.

Figure S3. The phase and amplitude of core clock gene rhythms in central tissues is influenced by photoperiod. Double-plotted rhythms in *Per1*, *Per2, Cry1, and Cry2* mRNA expression were measured with qRT-PCR for the cerebellum (CB), hippocampus (HIP), olfactory bulb (OB), and septum (SEP) under LD12:12 and LD20:4. White and black bars on the abscissa represent lighting conditions. n = 3/timepoint/photoperiod. Cosinor analyses are in Table S3.

Figure S4. Representative, background-subtracted images representing PER2 expression in the hippocampus of mice held under LD12:12 and LD20:4 measured with immunohistochemistry. Data are summarized in Figure 5 and Table S4.

Figure S5. Representative, background-subtracted images representing PER2 expression in the septum of mice held under LD12:12 and LD20:4 measured with immunohistochemistry. Data are summarized in Figure 5 and Table S4.

Figure S6. Representative, background-subtracted images representing PER2 expression in the SCN of mice held under LD12:12 and LD20:4 measured with immunohistochemistry. Data are summarized in Figure 6 and Table S4, also see [21].

Figure S7. Representative, background-subtracted images representing AVP expression in the SCN of mice held under LD12:12 and LD20:4 measured with immunohistochemistry. Data are summarized in Figure 6 and Table S4.

Table S1. PER2::LUC rhythms from non-SCN tissues collected at two different times of day.

	Peak Time		Period		Amplitude		Damping	
	**ZT02**	**ZT10**	**ZT02**	**ZT10**	**ZT02**	**ZT10**	**ZT02**	**ZT10**
**Not Reset by Dissection**								
ADR	17.9 ± 0.5	16.8 ± 1.0	22.4 ± 0.7	22.6 ± 1.7	51 ± 7	41 ± 4	1.9 ± 0.4	1.4 ± 0.8
APIT	19.5 ± 0.5	19.1 ± 0.3	25.1 ± 0.2	25.1 ± 0.1	29 ± 4	27 ± 5	2.8 ± 0.3	4.4 ± 0.3*
BAT	18.8 ± 0.7	17.9 ± 0.5	23.3 ± 1.0	25.0 ± 1.1	37 ± 9	60 ± 12	0.9 ± 0.1	2.6 ± 0.9
EWAT	19.5 ± 0.6	19.7 ± 0.4	24.3 ± 1.0	25.1 ± 0.1	143 ± 23	208 ± 42	0.9 ± 0.3	1.2 ± 0.2
IWAT	18.9 ± 0.4	18.2 ± 0.8	23.6 ± 0.5	22.7 ± 0.3	298 ± 58	316 ± 61	0.9 ± 0.1	1.5 ± 0.3
KID	15.8 ± 0.1	15.8 ± 0.1	28.1 ± 0.7	30.3 ± 0.7	29 ± 4	21 ± 5	2.5 ± 0.5	2.7 ± 1.0
LNG	17.6 ± 0.4	16.6 ± 0.4	25.5 ± 0.3	24.9 ± 0.6	73 ± 4	79 ± 9	4.8 ± 1.4	2.9 ± 0.8
MWAT	19.4 ± 1.4	17.6 ± 1.5	27.5 ± 3.2	28.9 ± 3.0	333 ± 50	282 ± 52	1.4 ± 0.2	1.8 ± 0.1
PIN	17.0 ± 0.6	18.2 ± 0.4	24.8 ± 0.6	24.2 ± 0.2	16 ± 3	27 ± 3*	1.7 ± 0.2	2.7 ± 0.6
PPIT	17.2 ± 0.7	16.7 ± 0.2	23.0 ± 0.3	23.5 ± 0.2	30 ± 6	48 ± 7	4.5 ± 0.6	4.4 ± 1.3
RWAT	21.0 ± 0.6	21.2 ± 0.9	32.7 ± 4.4	21.6 ± 0.6	98 ± 17	131 ± 24	2.6 ± 1.8	2.6 ± 1.2
SPLN	18.3 ± 0.6	18.5 ± 0.5	23.0 ± 0.5	23.1 ± 0.3	44 ± 6	41 ± 8	2.6 ± 0.2	3.6 ± 0.3
THY	19.5 ± 0.2	18.9 ± 0.9	23.8 ± 0.3	24.4 ± 0.9	151 ± 48	90 ± 12	2.6 ± 0.2	2.6 ± 0.4
**Reset by Dissection**								
CORN	24.3 ± 0.7	14.7 ± 0.1**	23.3 ± 0.7	23.2 ± 0.6	19 ± 1	56 ± 9**	3.8 ± 1.1	0.9 ± 0.1*
ESO	16.6 ± 0.3	20.1 ± 0.5*	23.8 ± 0.3	22.7 ± 0.8	76 ± 18	31 ± 10*	4.0 ± 0.5	2.5 ± 0.5
LIV	12.7 ± 0.1	15.7 ± 0.4**	19.0 ± 0.4	21.5 ± 0.5*	89 ± 11	31 ± 9**	1.1 ± 0.2	1.4 ± 0.2
RET	27.5 ± 0.9	17.8 ± 0.4**	24.9 ± 1.2	23.8 ± 0.3	8 ± 1	12 ± 2	0.6 ± 0.2	2.9 ± 1.3*
RPE	24.3 ± 1.3	17.1 ± 0.2*	25.3 ± 0.6	24.9 ± 0.3	12 ± 2	29 ± 2**	2.5 ± 0.7	1.9 ± 0.1

* $p < 0.05$, ** $p < 0.005$

Table S2. Photoperiodic changes in PER2::LUC rhythms of non-SCN tissues.

	Peak Time		Period		Amplitude		Damping	
	**LD12:12**	**LD20:4**	**LD12:12**	**LD20:4**	**LD12:12**	**LD20:4**	**LD12:12**	**LD20:4**
ADR	17.1 ± 0.3	14.2 ± 0.5**	21.9 ± 1.3	21.7 ± 0.6	47 ± 4	54 ± 7	4.7 ± 1.9	3.3 ± 1.0
APIT	14.1 ± 0.4	10.7 ± 0.7**	24.7 ± 0.5	25.7 ± 0.3	33 ± 8	32 ± 5	3.5 ± 0.3	2.6 ± 0.2*
BAT	20.2 ± 1.0	14.2 ± 1.6*	24.1 ± 0.5	24.7 ± 0.5	55 ± 19	110 ± 35	1.7 ± 0.3	1.1 ± 0.1*
EWAT	20.3 ± 0.3	17.6 ± 0.4*	23.6 ± 0.3	25.2 ± 0.3*	295 ± 53	559 ± 74*	1.7 ± 0.1	1.0 ± 0.1**
IWAT	18.1 ± 0.6	16.8 ± 0.5	23.2 ± 0.4	23.9 ± 0.3	281 ± 69	481 ± 69*	1.4 ± 0.3	1.1 ± 0.1
KID	15.6 ± 0.3	16.3 ± 0.7	25.4 ± 0.4	27.2 ± 0.5*	14 ± 2	10 ± 1	1.6 ± 0.3	1.8 ± 0.2
LNG	16.9 ± 0.6	15.7 ± 0.4	24.2 ± 0.2	24.5 ± 0.5	68 ± 12	47 ± 8	2.4 ± 0.5	1.7 ± 0.1
MWAT	18.5 ± 0.6	15.5 ± 0.8*	24.0 ± 0.3	23.9 ± 0.3	337 ± 51	614 ± 91*	1.6 ± 0.1	1.4 ± 0.1
PIN	17.7 ± 0.4	15.5 ± 0.4*	24.8 ± 0.3	25.5 ± 0.4	43 ± 8	27 ± 4	1.5 ± 0.1	1.2 ± 0.1
PPIT	16.8 ± 0.1	14.0 ± 0.2**	23.8 ± 0.1	23.3 ± 0.1*	61 ± 9	82 ± 9	3.0 ± 0.1	2.8 ± 0.2
RWAT	19.8 ± 0.4	18.5 ± 1.1	21.9 ± 0.1	23.2 ± 0.2**	186 ± 28	243 ± 29	3.8 ± 0.8	1.6 ± 0.3*
SPLN	19.7 ± 0.5	18.7 ± 0.4	23.6 ± 0.3	24.7 ± 0.7	25 ± 5	16 ± 2*	1.6 ± 0.2	1.5 ± 0.1
THY	20.7 ± 0.7	18.8 ± 0.3*	25.2 ± 0.9	26.8 ± 0.6	51 ± 12	41 ± 9	1.3 ± 0.1	1.2 ± 0.2

* $p < 0.05$, ** $p < 0.005$

Table S3. Photoperiodic changes in clock gene rhythms of non-SCN tissues.

	Rhythmicity		Center of Gravity		Amplitude	
	**LD12:12**	**LD20:4**	**LD12:12**	**LD20:4**	**LD12:12**	**LD20:4**
Per1						
CB	p < 0.005	p = 0.80	17.2 ± 0.5	15.9 ± 0.6	2.0 ± 0.2	0.5 ± 0.1**
HIP	p < 0.0005	p < 0.05	16.4 ± 0.5	12.4 ± 0.5**	2.4 ± 0.2	1.0 ± 0.1**
OB	p < 0.01	p = 0.20	19.0 ± 0.5	17.9 ± 0.5	3.6 ± 0.3	1.3 ± 0.1**
SEP	p < 0.005	p = 0.16	14.2 ± 0.6	15.5 ± 0.6	3.0 ± 0.3	1.4 ± 0.1**
Per2						
CB	p < 0.0001	p = 0.40	18.4 ± 0.4	13.9 ± 0.6*	2.9 ± 0.2	0.8 ± 0.1*
HIP	p < 0.0001	p < 0.0001	17.9 ± 0.5	14.1 ± 0.5**	2.4 ± 0.2	1.5 ± 0.1**
OB	p < 0.005	p = 0.08	18.5 ± 0.4	17.3 ± 0.4	4.2 ± 0.3	1.1 ± 0.1**
SEP	p < 0.05	p = 0.06	14.4 ± 0.6	13.8 ± 0.6	2.4 ± 0.2	0.9 ± 0.1**
Cry1						
CB	p = 0.08	p = 0.85	17.8 ± 0.6	18.4 ± 0.6	1.6 ± 0.1	0.6 ± 0.1**
HIP	p < 0.0005	p = 0.12	17.5 ± 0.5	14.7 ± 0.6**	2.8 ± 0.2	0.6 ± 0.1**
OB	p < 0.05	p = 0.16	20.8 ± 0.6	20.9 ± 0.5	2.1 ± 0.2	0.4 ± 0.1**
SEP	p = 0.08	p = 0.28	12.0 ± 0.7	15.1 ± 0.6**	1.6 ± 0.1	1.5 ± 0.2
Cry2						
CB	p = 0.08	p = 0.90	18.3 ± 0.6	19.7 ± 0.6	1.5 ± 0.2	1.0 ± 0.1**
HIP	p < 0.005	p = 0.12	16.5 ± 0.6	12.7 ± 0.6**	1.6 ± 0.2	0.7 ± 0.1**
OB	p < 0.005	p < 0.05	19.2 ± 0.6	19.6 ± 0.5	3.3 ± 0.3	0.9 ± 0.1**
SEP	p < 0.05	p = 0.08	14.5 ± 0.6	12.7 ± 0.6	2.5 ± 0.2	1.2 ± 0.1**

* p < 0.05, ** p < 0.005

For cosinor analyses, df = (2,21); For photoperiodic comparisons, df = 46

Table S4. Photoperiodic changes in protein rhythms of SCN and non-SCN tissues.

	Rhythmicity		Center of Gravity		Amplitude	
	**LD12:12**	**LD20:4**	**LD12:12**	**LD20:4**	**LD12:12**	**LD20:4**
**HIP**						
CA1 PER2	p < 0.0005	p < 0.0005	3.0 ± 0.4	24.8 ± 0.6*	43.9 ± 3.9	23.4 ± 2.6*
CA3 PER2	p < 0.0005	p < 0.05	3.8 ± 0.6	24.5 ± 0.4*	41.5 ± 2.7	35.6 ± 3.0
DG PER2	p < 0.0005	p < 0.001	4.9 ± 0.4	24.4 ± 0.4*	37.6 ± 3.5	24.0 ± 2.6
Hilus PER2	p < 0.01	p = 0.07	3.2 ± 0.6	24.4 ± 0.6*	15.1 ± 0.7	7.1 ± 1.1*
**SEP**						
LS PER2	p < 0.0005	p = 0.34	6.4 ± 0.7	4.2 ± 0.7	7.7 ± 0.6	5.0 ± 0.5
MS PER2	p < 0.01	p = 0.12	6.1 ± 0.7	5.1 ± 0.7	9.9 ± 0.8	6.3 ± 0.7
**SCN**						
Shell PER2	p < 0.0005	p < 0.0005	13.7 ± 0.3	11.2 ± 0.5*	10.7 ± 1.0	10.9 ± 0.7
Core PER2	p < 0.0005	p < 0.0005	13.0 ± 0.4	4.2 ± 0.5*	5.9 ± 0.6	5.7 ± 0.4
Shell AVP	p < 0.005	p = 0.11	14.0 ± 0.6	13.2 ± 0.6	5.8 ± 0.6	2.1 ± 0.4*

* $p < 0.05$;

For cosinor analyses, df = (2,21); For photoperiodic comparisons, df = 46

Table S5. Primers used for qRT-PCR

Gene	Forward Primer Sequence	Reverse Primer Sequence	Product Size, bp	Gene Accession
Per1	TGAGGAGCCAGAGAGGAAAG	GCAGTGTAGGAGGAGGAGGA	141	NM_011065
Per2	GAAAGCTGTCACCACCATAGAA	AACTCGCACTTCCTTTTCAGG	186	NM_011066
Cry1	CCTTATCTCCGCTTTGGTTG	CACAGGAGTTGCCCATAAAGA	119	NM_007771
Cry2	CTCCTGCCGCCTCTTCTAC	CCTCCATTCGGTCAAACCT	152	NM_009963
P0	CCGCCTGGTTCTCCTATAAAAGGCA	CGATGTCACTCCAACGAGGACGC	78	NM_007475
Bactin	CACCCGCGAGCACAGCTTCT	TTTGCACATGCCGGAGCCGT	118	NM_007393
18S	GTAACCCGTTGAACCCCATT	CCATCCAATCGGTAGTAGCG	151	NM_003278
Cyclophilin	GCTGGCGACTTCACCAACCACA	TGGACAGGACACCTGGCCCC	109	NM_134084
Gapdh	AAACACGGGGGCAATGAGTG	TTGGCATTGTGGAAGGGCTC	152	NM_007475

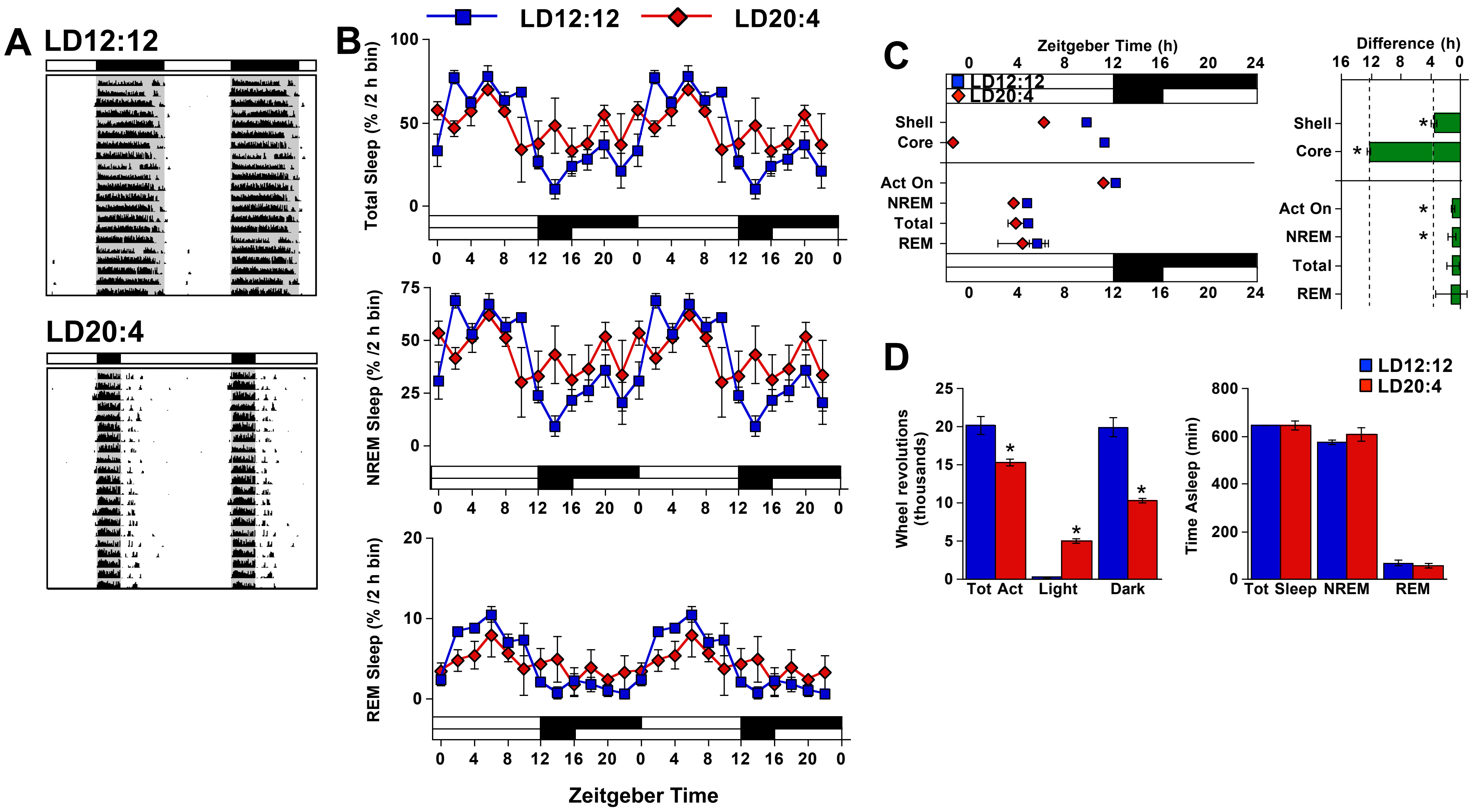

A LD12:12
LD20:4
B
LD12:12
LD20:4
Total Sleep (% /2 h bin)
NREM Sleep (% /2 h bin)
REM Sleep (% /2 h bin)
Zeitgeber Time
C
Zeitgeber Time (h)
LD12:12
LD20:4
Shell
Core
Act On
NREM
Total
REM
Difference (h)
D
Wheel revolutions (thousands)
Tot Act
Light
Dark
Time Asleep (min)
Tot Sleep
NREM
REM
LD12:12
LD20:4

Figure S1

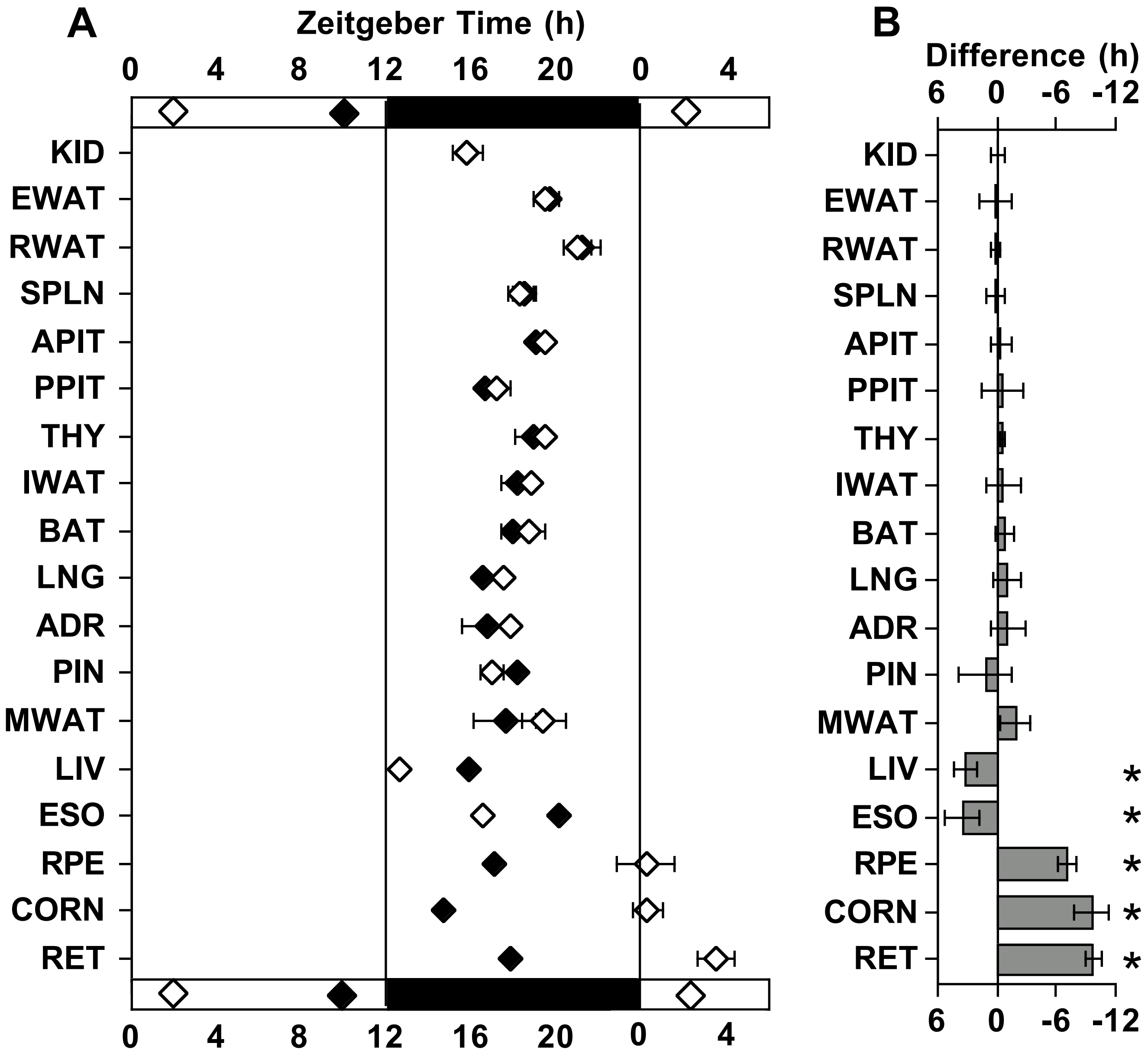
A
Zeitgeber Time (h)
0 4 8 12 16 20 0 4
KID
EWAT
RWAT
SPLN
APIT
PPIT
THY
IWAT
BAT
LNG
ADR
PIN
MWAT
LIV
ESO
RPE
CORN
RET
B
Difference (h)
6 0 -6 -12
*
*
*
*
*

Figure S2

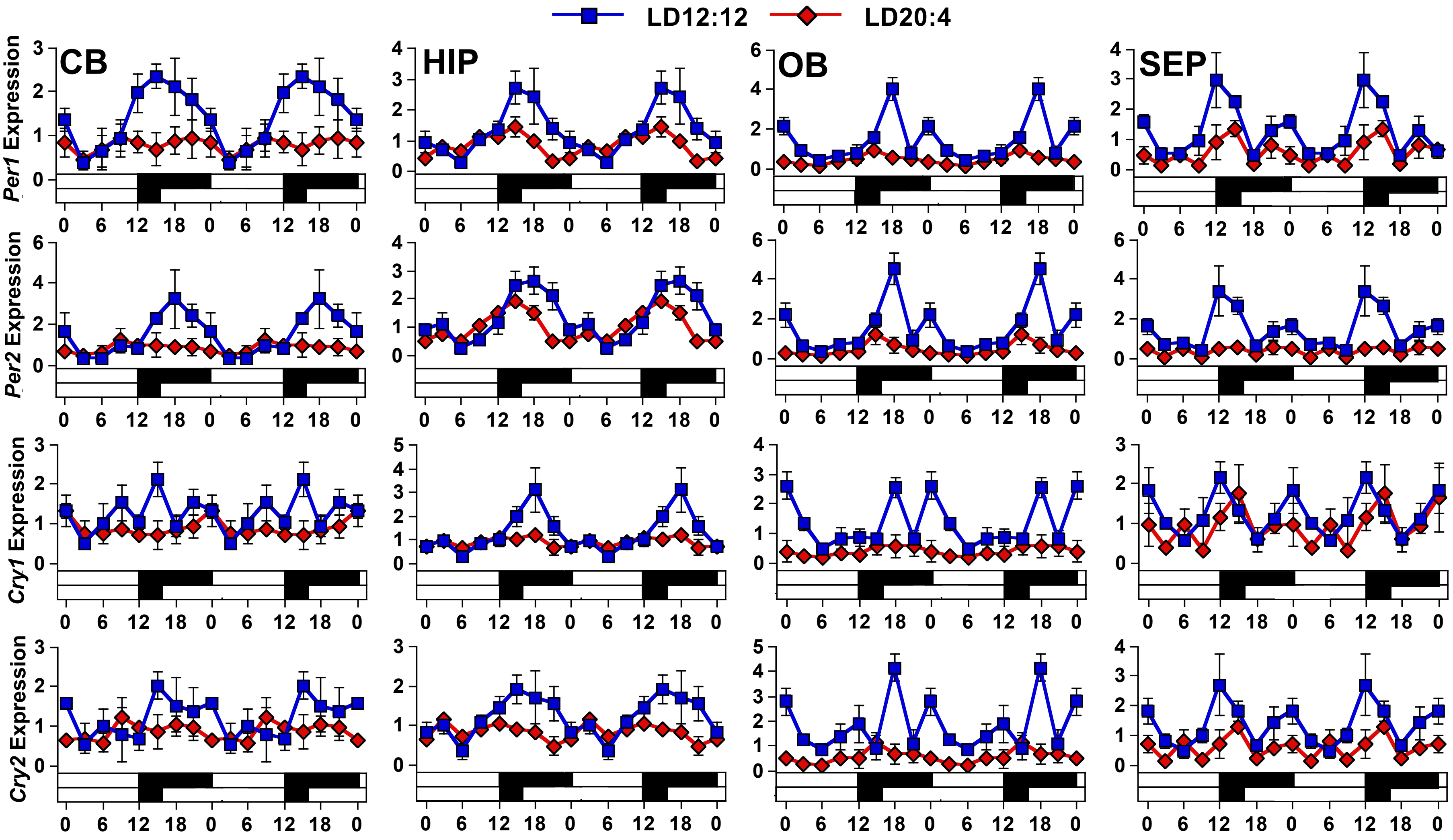
LD12:12
LD20:4
CB
HIP
OB
SEP
Per1 Expression
Per2 Expression
Cry1 Expression
Cry2 Expression
0 6 12 18 0 6 12 18 0

Figure S3

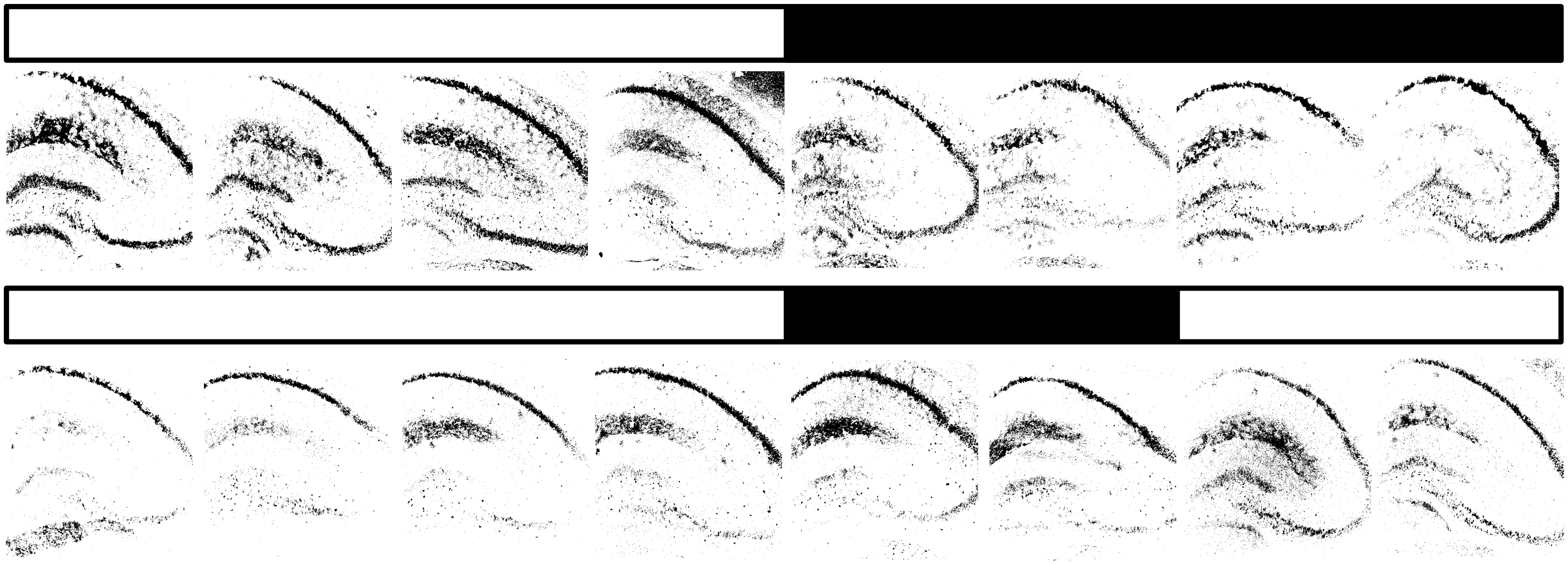

Figure S4

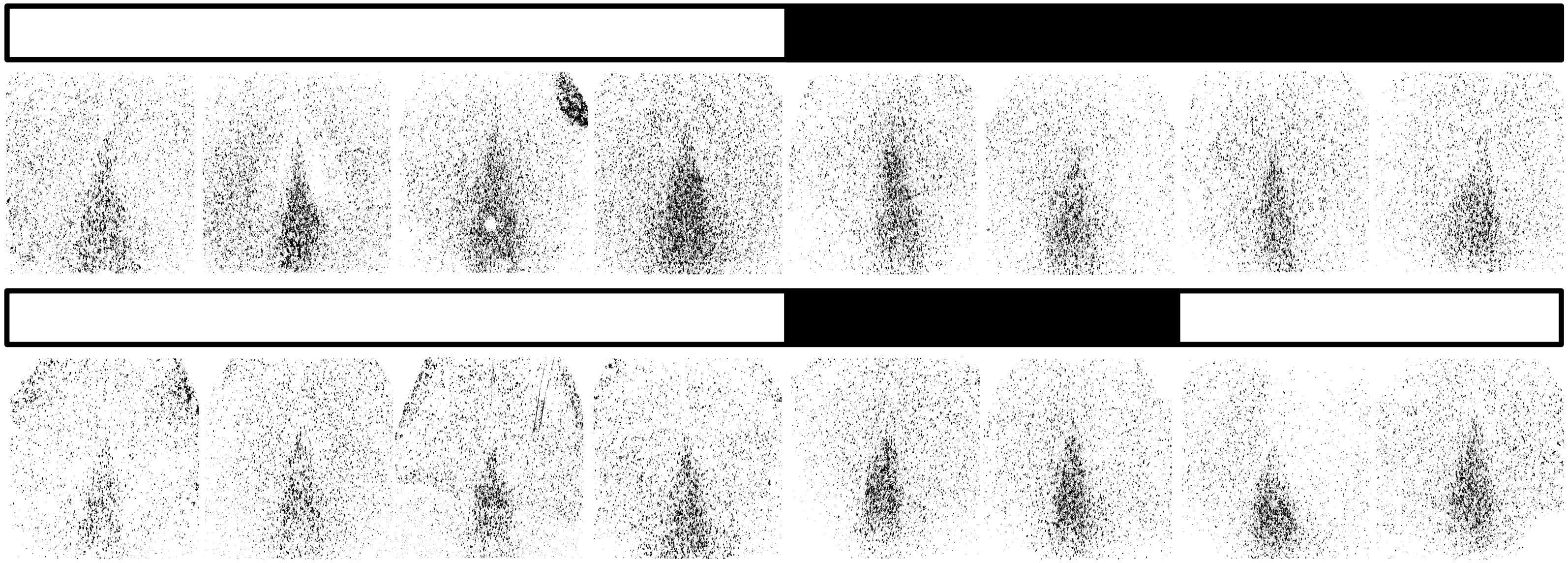

Figure S5

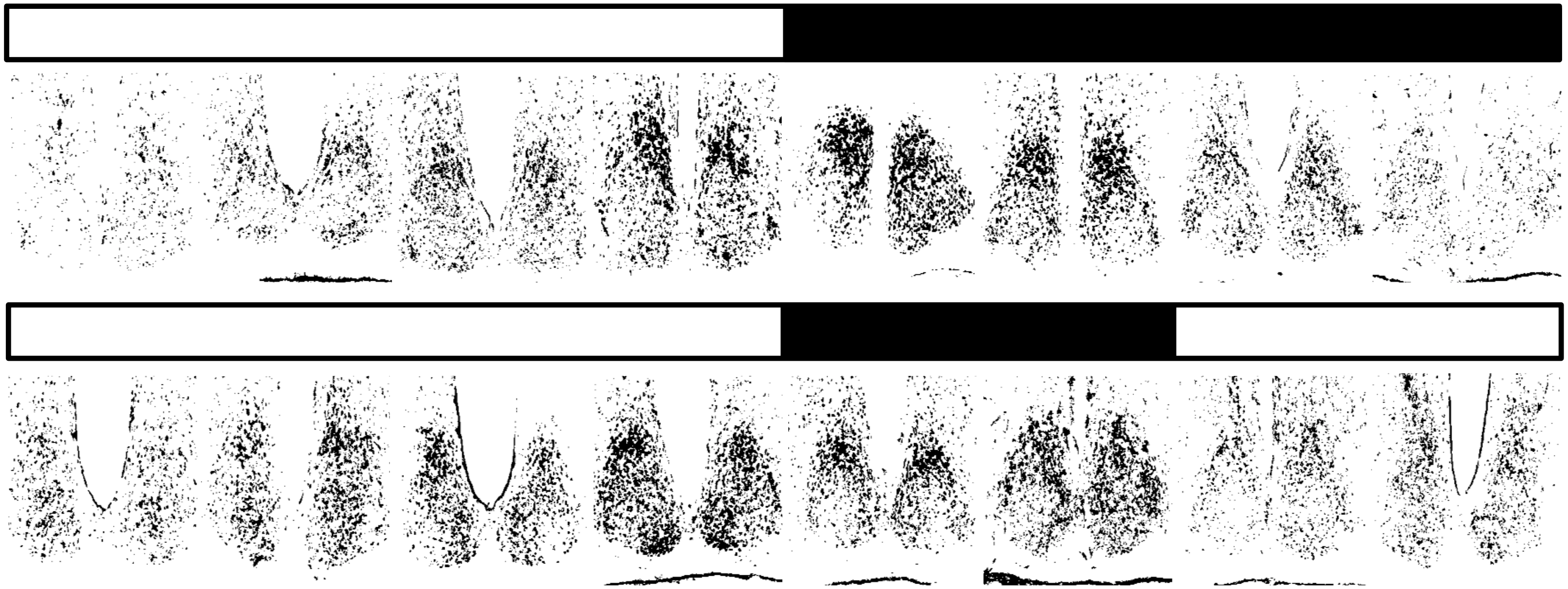

Figure S6

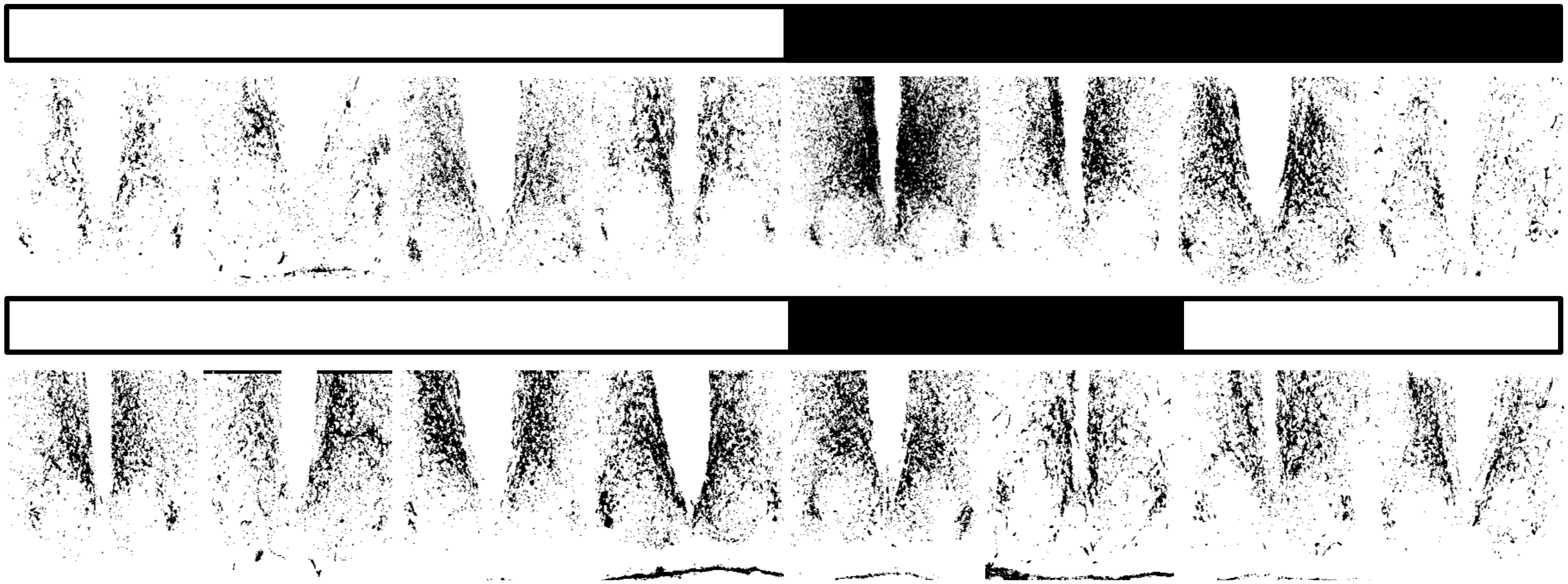

Figure S7
